# *Salmonella enterica* Typhimurium infection causes metabolic changes in chicken muscle involving AMPK, fatty acid and insulin/mTOR signaling

**DOI:** 10.1186/1297-9716-44-35

**Published:** 2013-05-17

**Authors:** Ryan J Arsenault, Scott Napper, Michael H Kogut

**Affiliations:** 1 United States Department of Agriculture, Agricultural Research Service, SPARC, College Station, TX, 77845, USA; 2 Department of Biochemistry, University of Saskatchewan, Saskatoon, SK, S7N 5E3, Canada

## Abstract

*Salmonella enterica* serovar Typhimurium (*Salmonella* Typhimurium) infection of chickens that are more than a few days old results in asymptomatic cecal colonization with persistent shedding of bacteria. We hypothesized that while the bacterium colonizes and persists locally in the cecum it has systemic effects, including changes to metabolic pathways of skeletal muscle, influencing the physiology of the avian host. Using species-specific peptide arrays to perform kinome analysis on metabolic signaling pathways in skeletal muscle of *Salmonella* Typhimurium infected chickens, we have observed key metabolic changes that affected fatty acid and glucose metabolism through the 5'-adenosine monophosphate-activated protein kinase (AMPK) and the insulin/mammalian target of rapamycin (mTOR) signaling pathway. Over a three week time course of infection, we observed changes in the phosphorylation state of the AMPK protein, and proteins up and down the pathway. In addition, changes to a large subset of the protein intermediates of the insulin/mTOR pathway in the skeletal muscle were altered by infection. These changes occur in pathways with direct effects on fatty acid and glucose metabolism. This is the first report of significant cellular metabolic changes occurring systemically in chicken due to a *Salmonella* infection. These results have implications not only for animal production and health but also for the understanding of how *Salmonella* infection in the intestine can have widespread, systemic effects on the metabolism of chickens without disease-like symptoms.

## Introduction

*Salmonella* are enteric bacteria, and salmonellosis is a major infectious disease around the world. A number of *Salmonella* serovars are responsible for human disease with symptoms ranging from gastroenteritis to sepsis [1]. *Salmonella* are important zoonotic agents that infect a wide variety of animal species. *Salmonella enterica* serovar Typhimurium (*Salmonella* Typhimurium) is a rod-shaped, flagellated, aerobic, Gram-negative bacterium. *Salmonella* Typhimurium is infectious to humans and can cause severe gastro-intestinal pathology and typhoid fever.

*Salmonella* Typhimurium infection of day-old chickens can result in severe inflammatory responses and intestinal pathology [2]; however, *Salmonella* Typhimurium infection in older chickens does not cause overt pathogenic symptoms [3]. The lack of pathology observed in week-old birds infected with *Salmonella* Typhimurium may be due to a lack of a pro-inflammatory cytokine response and a release of TGF-β4 in these older birds [4]. Despite the lack of obvious disease symptoms and pathology, it is clear that profound changes are occurring in the chicken host, and many of these changes have not been fully characterized.

Peptide arrays for kinome analysis have been utilized by a number of groups to study cellular signaling, ranging from the study of cancer [5] to bacterial ligands [6]. A more recent advancement has been the use of species-specific peptide arrays for kinomic study of less common research species such as bovine and ovine [7]. In addition, the use of these species-specific peptide arrays has branched out beyond the original host-bacterial [8] and host-viral [9] interaction studies to consider diverse biological phenomena such as prion biology [10]. As so much cellular function is controlled by protein phosphorylation, it is possible to consider other biological functions besides the standard immunity or cell growth/cycle regulation. Here, we introduce a chicken species-specific peptide array purposely designed for the study of cellular metabolic signaling. To our knowledge, this is the first use of a peptide array for kinomic study of this species and this cellular function.

The fact that alterations to the intestinal environment affect animal growth has been known for many years. Growth-promoting antibiotics are thought to carry out their function through reduced local inflammation, reduced intestinal size and, most importantly, reduced competition for nutrients in the small intestine [11]. Studies have shown that the population makeup of the gut microbiota is altered by the administration of growth promoting antibiotics [12]. It is reasonable to assume that an infection of the intestinal tract would influence metabolism as well, since it would likely compete with other bacterial species in the gut and possibly affect inflammation: two processes known from antibiotic studies to affect animal growth [11].

The 5'-adenosine monophosphate-activated protein kinase (AMPK) enzyme is a key metabolic energy homeostasis regulator in avian species [13]. One of its roles is to monitor the ratio of AMP:ATP and alter metabolic processes accordingly. In addition, AMPK can receive signals of cellular energy state and send corresponding signals, via phosphorylation, that can affect cellular processes such as glycolysis/gluconeogenesis, protein synthesis, fatty acid synthesis and fatty acid oxidation. AMPK activity is known to be affected by changes in the gut microbiota though the mechanism is unclear [14].

The insulin signaling pathway and mammalian target of rapamycin (mTOR) signaling are extensively linked and display significant overlap, so much so that it is referred to as the insulin/mTOR signaling pathway [15]. mTOR signaling plays an important role in cellular metabolism and has been linked to obesity and diabetes. Experimentally altered gut microbiota in mice has led to obesity, metabolic syndrome and insulin resistance: disorders related to the insulin/mTOR signaling pathway [16]. While there appear to be significant differences between mammalian and avian responses to insulin, to the point where chickens have been described as insulin resistant [17], the key constituents of the insulin/mTOR signaling pathway are similar. Despite the differential sensitivity to insulin, evidence suggests that chickens still respond to insulin; despite questions on the exact nature of the pathway between the insulin receptor and Akt, all of the members of the pathway appear to be present in the chicken proteome [18]. The mTOR pathway appears well conserved between mammals and chickens, though the level of conservation among peptides upstream remains a question. Based on the evidence, it is possible to assume that the mTOR pathway may play a role in abnormal metabolism that is similar to that seen in humans or mice. Such similarity seems likely, especially considering how AMPK has been extensively studied in avian species and how AMPK is involved in mTOR signaling.

In this study, we used a chicken-specific global metabolic peptide array to study the changes in skeletal muscle metabolism over time following *Salmonella* Typhimurium infection. We hypothesized that while the bacterium colonizes and persists locally in the cecum, it has systemic effects influencing the metabolism of skeletal muscle. While this may not be a direct immunological effect, a disruption in metabolism and energy resources can have a profound effect on the health of the host. Our results clearly point to alterations in AMPK phosphorylation and activity as well as significant disruptions in the insulin/mTOR signaling pathway involving numerous pathway intermediates. We subsequently confirmed these results through the use of the complementary technique of antibody microarray analysis.

## Materials and methods

### Ethics statement

These studies were approved by the Animal Care and Use Committee (ACUC) at the Southern Plains Agricultural Research Center, Agricultural Research Service, United States Department of Agriculture (ACUC #2012007), which meets all federal requirements as defined in the Animal Welfare Act, the Public Health Service Policy, and the Humane Care and Use of Laboratory Animals.

### Animal hatch and infection

Fifty 17-day-old broiler eggs, which had not been vaccinated or otherwise treated, were procured from Sanderson Farms (Bryan, TX, USA). These eggs were incubated at 37.2°C and 92% humidity for 3 days before hatching. Forty of 50 eggs hatched after 3 days. Five days post-hatch, half were orally infected with 1 × 10^5^ cfu of *Salmonella* Typhimurium that had been grown in tryptic soy broth with 20 ug/mL novobiocin and 25 ug/mL nalidixic acid. Control and infected animals were kept in separate isolation rooms, and cross-contamination was strictly controlled for. At 24 h, 96 h, 1 week and 3 weeks post infection, animals were sacrificed and samples were collected.

### Sample collection

At 24 h, 96 h, 1 week and 3 weeks post infection, birds were sacrificed by CO_2_ asphyxiation and muscle samples were collected. Muscle samples from the thigh were removed and immediately flash frozen in liquid nitrogen to preserve kinase enzymatic activity. Samples were taken from liquid nitrogen and transferred to a −80°C freezer until further experimental procedures were conducted.

### Peptide arrays

At each of the 4 time points and under each condition (infected and uninfected), 3 muscle samples from 3 different animals were taken from storage for analysis (24 samples total). Muscle samples were weighed to obtain a consistent 40 mg samples for the array protocol. Samples were homogenized by a hand-held Qiagen TissueRuptor (Valencia, CA, USA) in 100 uL of lysis buffer (20 mM Tris–HCl pH 7.5, 150 mM NaCl,1 mM EDTA, 1 mM Ethylene glycol tetraacetic acid (EGTA), 1% Triton X-100, 2.5 mM sodium pyrophosphate, 1 mM Na3VO4, 1 mM NaF, 1 μg/mL leupeptin, 1 g/mL aprotinin and 1 mM Phenylmethylsulphonyl fluoride (all products from Sigma Aldrich (St. Louis, MO, USA), unless indicated)). Following homogenization, the peptide array protocol was carried out as per Jalal et al. [7] with alterations described in Arsenault et al. [8].

### Antibody arrays

The mTOR Signaling Phospho-Specific Antibody Array and antibody array assay kit were procured from Full Moon BioSystems (Sunnyvale, CA, USA). This technique was used as an alternative to procuring phosphospecific antibodies individually and performing several western blot assays. Protocol was carried out as per manufacturer’s instructions with the following alteration to the homogenization step: instead of using the bead and vortex homogenization indicated in the kit, the hand-held Qiagen TissueRuptor was used.

### Data analysis

Data normalization and PCA analysis was performed for both the peptide and antibody microarrays as per Li et al. [19]. This consistent analysis method facilitated a more direct comparison between the two distinct array datasets and allowed for a statistically robust analysis of the phosphorylation events being measured. Geneontology (GO) and Kyoto Encyclopedia of Genes and Genomes (KEGG) pathway analysis was performed by uploading the statistically significant peptide lists to the Search Tool for the Retrieval of Interacting Genes (STRING) [20].

## Results

Non-vaccinated, non-treated broiler eggs were procured and hatched. Five days post-hatch, half the chicks were orally infected with 1 × 10^5^ cfu of *Salmonella* Typhimurium. Control and infected animals were kept in separate isolation rooms, and cross-contamination was strictly controlled for. At 24 h, 96 h, 1 week and 3 weeks post infection, animals were sacrificed and samples collected. Infection status was confirmed by *Salmonella* Typhimurium culturing of cecal contents and feces from each animal with and without enrichment. Cultures confirmed that the infected group displayed *Salmonella* infection throughout the experiment, while *Salmonella* was not isolated from animals in the control group. Samples were snap-frozen in liquid nitrogen as quickly as possible to preserve enzymatic activity.

**Figure 1:**
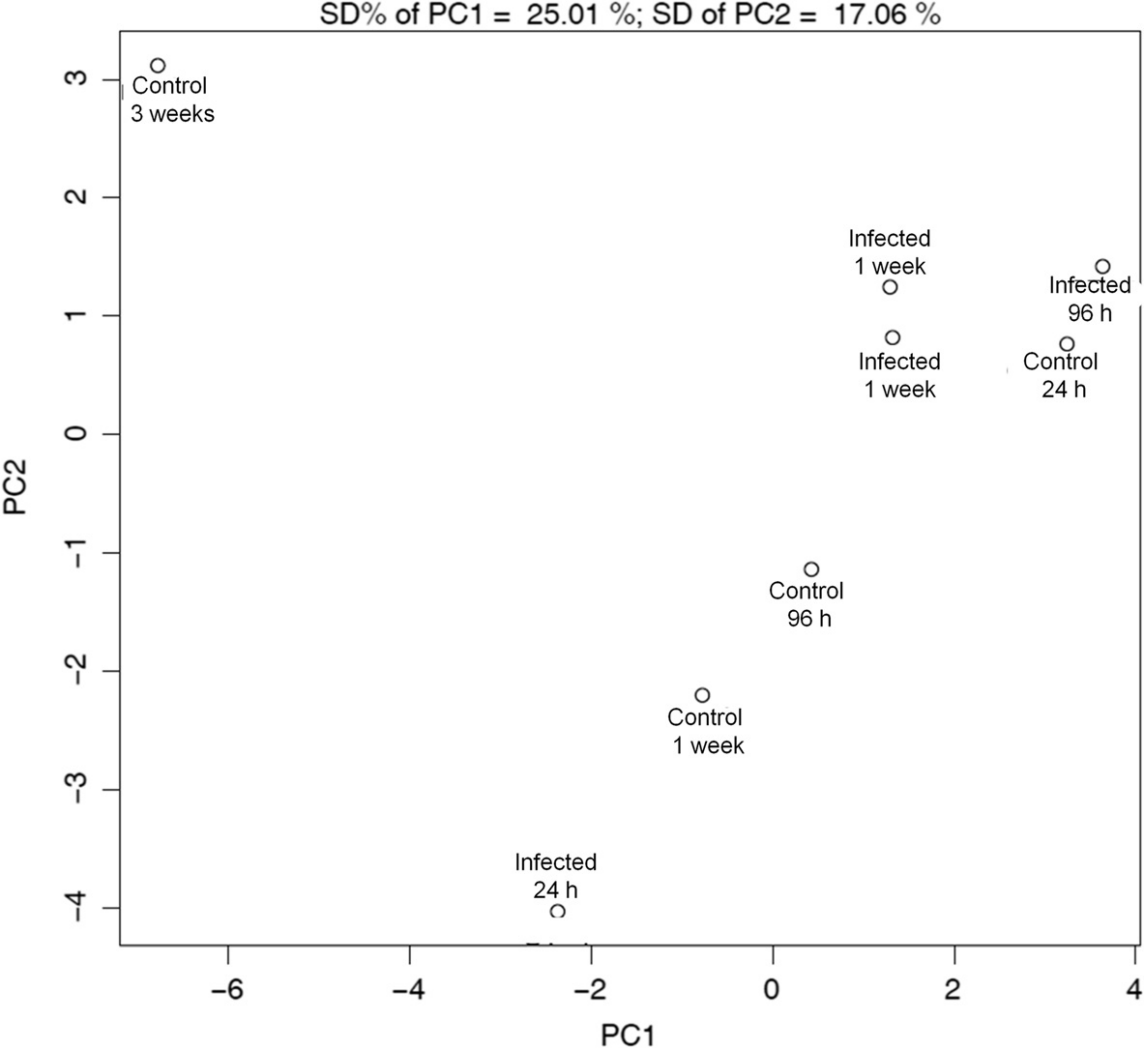
**Principal component analysis of averaged peptide array results.** For each condition, samples were collected from 3 animals and peptide arrays were performed. Following normalization, data from each treatment was averaged together to generate a representative data set for each condition. Then PCA was performed on each of these representative data sets. Each data point represents 3 biologically distinct animals.

Chicken-specific peptide arrays designed for the study of chicken metabolic signaling pathways were used to analyze 24 thigh muscle samples. The results from the three animals from each condition (infected and uninfected) and time point were averaged together to provide a representative result (Additional file 1). PCA was conducted on the resultant data, and distinct patterns of clustering were observed (Figure 1). The infected birds from the later three time points (96 h, 1 week and 3 week) clustered together, while the 24 h post-infection birds were separate. The control data at each of the time points did not cluster in any appreciable pattern. This was not unexpected, as very large metabolic changes would be occurring within these animals as they grew from days-old chicks to weeks-old birds, making the metabolic patterns at these time points very distinct.

To account for any changes in phosphorylation state that were not due to the infection, the results at each time point were corrected using their respective time-matched controls. All peptides that showed statistically significant differential phosphorylation (*p* < 0.05) for each time point were input into the Search Tool for the Retrieval of Interacting Genes (STRING) database [20]. Using STRING functionality, Geneontology (GO) results for biological processes and molecular functions, as well as Kyoto Encyclopedia of Genes and Genomes (KEGG) pathway results were generated from each dataset.

The KEGG pathway results generated from STRING showed a large number of pathways implicated by the data at a statistically significant level (*p* < 0.05 false discovery rate (FDR) corrected) (Table 1). Of particular interest were those pathways that showed statistically significant changes at multiple time points over the course of the study. These pathways are shown in bold in Table 1. Of note are the large number of metabolic and immunologic pathways that have been altered by the infection. These pathways included Adipocytokine signaling, Fc receptor pathway, glycolysis/gluconeogenesis, insulin signaling, mTOR pathway and Toll-like receptor signaling. The central signaling pathway MAPK also appeared affected by the infection at multiple time points. The changes in MAPK and metabolic signaling were likely the reasons why a number of cancer pathways were generated by the data. Though deregulation of MAPK intermediates and changes in metabolic regulation are hallmarks of cancer, cancer is very unlikely to be the result of *Salmonella* Typhimurium infection; thus these pathways can be disregarded, while the protein intermediates within the pathways themselves should be considered.

**Table 1:** KEGG Pathways generated by STRING

		**24 h**		**96 h**		**1 week**		**3 weeks**	
**Pathway ID**	**Pathway name**	**# Peptides**	***p*-value (FDR)**	**# Peptides**	***p*-value (FDR)**	**# Peptides**	***p*-value (FDR)**	**# Peptides**	***p*-value (FDR)**
hsa05221	Acute myeloid leukemia	4	0.0227	3	0.0344	2	0.228	3	0.0785
hsa04520	Adherens junction	4	0.0506	2	0.340	2	0.332	4	0.0302
**hsa04920**	**Adipocytokine signaling pathway**	**6**	**0.0023**		**N/S**	**4**	**0.0131**	**7**	**0.0003**
hsa04360	Axon guidance	6	0.0191	4	0.0432		N/S		N/S
hsa04662	B cell receptor signaling pathway	5	0.0123	3	0.0559		N/S	4	0.0297
hsa04020	Calcium signaling pathway	5	0.166		N/S	6	0.0095		N/S
hsa04062	Chemokine signaling pathway	4	0.469	5	0.0344	4	0.126		N/S
**hsa05220**	**Chronic myeloid leukemia**	**4**	**0.0452**	**4**	**0.0103**	**2**	**0.**318	**6**	**0.0012**
hsa05210	Colorectal cancer		N/S	4	0.0066		N/S	3	0.0969
hsa05213	Endometrial cancer	3	0.0976	4	0.0047		N/S		N/S
**hsa04012**	**ErbB signaling pathway**	**8**	**0.0001**	**7**	**0.0000**	**4**	**0.0167**	**5**	**0.0097**
**hsa04664**	**Fc epsilon RI signaling pathway**	**7**	**0.0006**	**5**	**0.0029**		**N/S**	**6**	**0.0018**
hsa04666	Fc gamma R-mediated phagocytosis	4	0.0979	4	0.0224		N/S	5	0.0148
**hsa04510**	**Focal adhesion**	**8**	**0.0123**	**6**	**0.0115**		**N/S**	**7**	**0.0154**
**hsa05214**	**Glioma**	**7**	**0.0001**	**3**	**0.0432**	**6**	**0.0001**	**5**	**0.0033**
**hsa00010**	**Glycolysis/Gluconeogenesis**	**5**	**0.0064**	**3**	**0.0432**	**3**	**0.0586**	**6**	**0.0008**
**hsa04912**	**GnRH signaling pathway**	**7**	**0.0015**	**5**	**0.0047**	**4**	**0.0245**	**5**	**0.0151**
hsa05410	Hypertrophic cardiomyopathy (HCM)	3	0.264		N/S	3	0.106	6	0.0018
**hsa04910**	**Insulin signaling pathway**	**13**	**0.0000**	**10**	**0.0000**	**7**	**0.0003**	**16**	**0.0000**
hsa04730	Long-term depression	4	0.0449	3	0.0537	4	0.0113		N/S
**hsa04010**	**MAPK signaling pathway**	**14**	**0.0001**	**13**	**0.0000**	4	0.318	**11**	**0.0011**
**hsa05218**	**Melanoma**	**4**	**0.0443**	**4**	**0.0095**	**4**	**0.0113**	**5**	**0.0043**
**hsa04150**	**mTOR signaling pathway**	**6**	**0.0004**	**3**	**0.0309**		**N/S**	**5**	**0.0018**
**hsa04722**	**Neurotrophin signaling pathway**	**6**	**0.0176**	**6**	**0.0020**	**5**	**0.0113**	**7**	**0.0018**
**hsa05223**	**Non-small cell lung cancer**	**5**	**0.0048**	**3**	**0.0362**		**N/S**	**4**	**0.0151**
**hsa05212**	**Pancreatic cancer**	**4**	**0.0418**	**3**	**0.0476**		**N/S**	**4**	**0.0227**
hsa05200	Pathways in cancer		N/S		N/S	6	0.0639	9	0.0151
hsa04914	Progesterone-mediated oocyte maturation	5	0.0176		N/S		N/S	5	0.0097
**hsa05215**	**Prostate cancer**	**5**	**0.0192**	**4**	**0.0173**	**4**	**0.0169**	**5**	**0.0107**
hsa04810	Regulation of actin cytoskeleton	6	0.104	6	0.0115		N/S	6	0.0591
**hsa05211**	**Renal cell carcinoma**	**4**	**0.0446**	**5**	**0.0018**		**N/S**	**4**	**0.0254**
hsa05222	Small cell lung cancer		N/S		N/S	2	0.397	6	0.0020
hsa04660	T cell receptor signaling pathway	6	0.0123	4	0.0344		N/S	3	0.332
**hsa04620**	**Toll-like receptor signaling pathway**	**5**	**0.0403**	**5**	**0.0058**		**N/S**	**6**	**0.0043**
hsa04270	Vascular smooth muscle contraction	5	0.0452	3	0.139		N/S	4	0.105
hsa04370	VEGF signaling pathway	6	0.0023		N/S		N/S	4	0.0302

Peptides that displayed a statistically significant change in phosphorylation state were input into the STRING database for each time point. Generated pathways that displayed a *p*-value of less than 0.05 (FDR corrected) are listed. Pathways that displayed statistical significance at three or more time points are shown in bold. N/S indicates that the pathway is Non-significant as no peptides were found within the pathway.

Using STRING GO analysis, the Molecular Function results generated for each time point were very similar. The most significant results related to adenyl phosphate moiety binding. Note that all the following results were FDR corrected. At 24 h, the top three results were adenyl ribonucleotide binding (*p*-value 5.77 × 10^-15^), adenyl nucleotide binding (*p*-value 5.77 × 10^-15^) and ATP binding (*p*-value 2.0 × 10^-14^). At 96 h, the top two results were adenyl ribonucleotide binding and adenyl nucleotide binding (both *p*-value 9.1 × 10^-5^). At 1 week, one of the top results was ATP binding (*p*-value 4.93 × 10^-4^). At 3 weeks, the top result was AMP-activated protein kinase activity (*p*-value 1 × 10^-5^), and the fourth and fifth results were adenyl ribonucleotide binding (*p*-value 4.8 × 10^-5^) and adenyl nucleotide binding (*p*-value 4.8 × 10^-5^) respectively. These results strongly implicated a central role for signaling events associated with the ATP, ADP and/or AMP levels, all of which play a role as energy currency in cells.

Again using the functionality in STRING, GO Biological Process results were generated from the data for each time point. Table 2 shows the top results for each time point excluding generic results that provide no relevant information. For example, processes such as “protein phosphorylation”, “enzyme linked receptor protein signaling pathway” and “intracellular signal transduction” were excluded. The results provided convincing evidence for some type of involvement of the insulin/glucose and fatty acid metabolic pathways in *Salmonella* Typhimurium infection. The Biological Process results, the KEGG pathway analysis and the molecular function results all provided evidence of altered metabolic activity upon infection.

**Table 2:** GO Biological processes

		**24 h**		**96 h**		**1 week**		**3 weeks**	
**GO ID**	**Term**	**# Peptides**	***p*-value (FDR)**	**# Peptides**	***p*-value (FDR)**	**# Peptides**	***p*-value (FDR)**	**# Peptides**	***p*-value (FDR)**
GO:0006112	energy reserve metabolic process	11	1.62E-07	8	3.71E-06		N/S	8	9.87E-06
GO:0008286	insulin receptor signaling pathway	11	2.43E-07	6	3.09E-04		N/S	9	3.49E-05
GO:0019217	regulation of fatty acid metabolic process	8	2.04E-06	4	2.75E-03	2	3.82E-01	8	2.27E-07
GO:0006091	generation of precursor metabolites and energy	13	1.59E-05	10	1.99E-05		N/S	9	8.88E-02
GO:0031325	positive regulation of cellular metabolic process	23	7.10E-04	18	2.11E-05		N/S	21	2.66E-07
GO:0005978	glycogen biosynthetic process	3	3.17E-03	2	3.44E-02	3	3.67E-03		N/A
GO:0016051	carbohydrate biosynthetic process	7	4.06E-04	3	1.62E-01	5	4.62E-03	7	5.64E-05
GO:0046320	regulation of fatty acid oxidation	6	1.32E-05	2	1.67E-01		N/S	7	1.08E-07

Peptides that displayed a statistically significant change (FDR corrected) in phosphorylation state were input into the STRING database for each time point. The top generated GO Biological Process results for each time point are listed, excluding generic results that are inherent to the analysis such as "protein phosphorylation", "enzyme linked receptor protein signaling pathway" and "intracellular signaling transduction". These provide no relevant data for analysis. N/S indicates that the pathway is Non-significant as no peptides were found within the pathway.”

**Figure 2:**
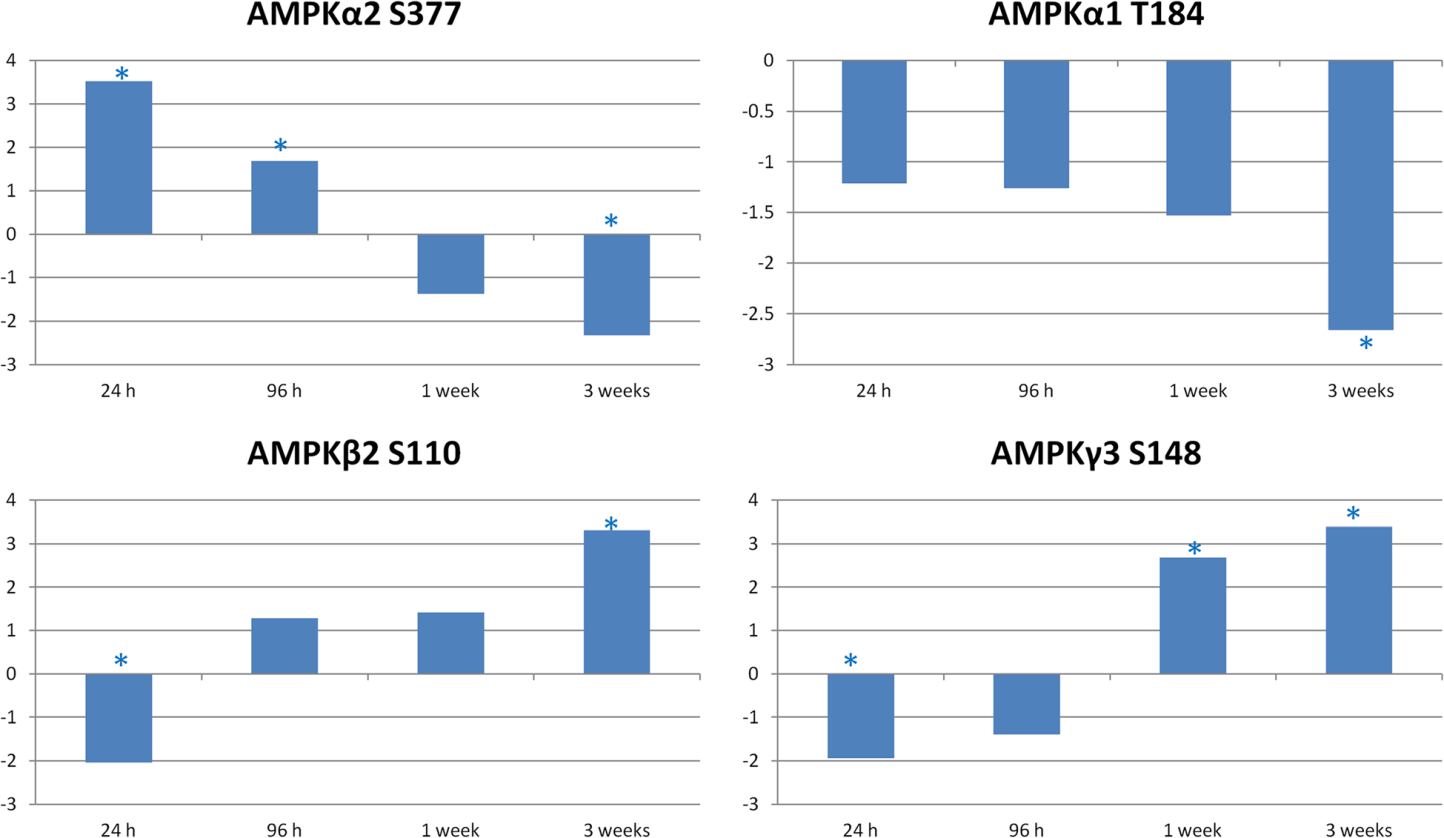
**AMPK related phosphorylation changes relative to control over time.** Comparison of *Salmonella* Typhimurium infected to uninfected muscle samples at each time point generate a phosphoylation fold change and a statistical significance value for each peptide on the array. The * indicates statistically significant difference in phosphorylation relative to control (*p* < 0.05).

Following the higher-order analysis, we considered the individual phosphorylation events and changes that occurred over the course of the study. A subset of AMPK-associated phosphorylation events were found to be significantly altered over the time course of the study. This enzyme functions as part of the energy homeostasis system in cells [13]. AMPK is activated by increased levels of AMP, which is a signal of low energy levels in the cell. AMPK has links to fatty acid metabolism, glucose uptake and insulin signaling. These pathways and processes were implicated in our previous analysis results. AMPK is made up of three subunits: an enzymatic kinase subunit (alpha) and two regulatory subunits (beta and gamma) [13]. Peptide array results indicated increased phosphorylation of AMPKα2 at S377 and decreased phosphorylation of AMPKβ2 S110 and AMPKγ3 S148 at 24 h post infection. At 3 weeks post infection, there was decreased phosphorylation of AMPKα2, as well as AMPKα1, with increased phosphorylation ofAMPKβ2 and AMPKγ3 (Figure 2).

Other phosphorylation events provided further evidence for the early activation of AMPK. Calcium/calmodulin-dependent protein kinase II (CaMK2) is known to affect AMPK phosphorylation [21]. Results showed that CaMK2 beta (the most active form of CaMK2) was phosphorylated 2.73 fold (*p* 0.005) over control at the 24 h time point. Also, AMPK is known to inhibit protein synthesis through phosphorylation-mediated inhibition of eukaryotic elongation factor-2 kinase (EF2K) at S398 but not S78 or S366 [22]. Peptide array results showed that EF2K S398 phosphorylation was increased 1.97 fold (*p* 0.017) at the 24 h time point. This was the same time point that showed the greatest AMPKα phosphorylation. The EF2K S78 peptide target was also present on the peptide array, but since this site phosphorylation is not affected by AMPK in vivo [22] it showed no significant phosphorylation at any time point as expected.

**Figure 3:**
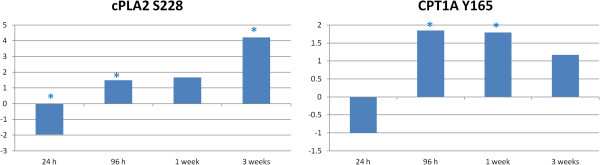
**Fatty acid related peptide changes relative to control over time.** Comparison of *Salmonella* Typhimurium infected to uninfected muscle samples at each time point generate a phosphoylation fold change and a statistical significance value for each peptide on the array. The * indicates statistically significant difference in phosphorylation relative to control (*p* < 0.05).

The later time points showed a reduced phosphorylation of AMPKα. One of the effects of reduced AMPK activity is a decrease in fatty acid oxidation and an increase in fatty acid and cholesterol synthesis [13]. We saw evidence that fatty acid metabolism was altered over the course of the study, as two of the GO biological processes implicated by the peptide array data were “regulation of fatty acid metabolic processes” and “regulation of fatty acid oxidation”, the latter only displayed significance at the 3 week time point. In a comparison of fat and lean birds, it has been shown that fat birds had reduced AMPK gene expression and reduced AMPKα phosphorylation [23]. This protein appears to be one of the key determinants of whether a bird is considered fat or lean. Besides AMPK, two peptides showed significant differential phosphorylation over numerous time points, are known to be involved in fatty acid processes and displayed increased phosphorylation over time (Figure 3). These were cytosolic phospholipase A2 (cPLA2), which has been implicated previously in fatty acid metabolism [24], and carnitine palmitoyltransferase 1A (CPT1A), which is related to fatty acid metabolism in mitochondria [25].

Acetyl-coenzyme A carboxylase 1 (ACC1) is an enzyme which is involved in long chain fatty acid synthesis. ACC1 activity is inhibited by phosphorylation at two sites: S80 (S79 in mice), which was not present on the peptide array, and S1263, ortholog of S1255 in chicken [26]. Peptide array results showed that there was a 2.18 fold change (*p* 0.042) increase in phosphorylation of S1255 of ACC1 at the 24 h time point. This change indicates early inhibition of fatty acid synthesis. At later time points, this phosphorylation site showed no significant phosphorylation indicating no inhibition of this enzyme and the potential for fatty acid synthesis. PPAR gamma co-factor 1 (PGC-1) is an enzyme which is present in adipose cells and skeletal muscle, and it coordinates fatty acid catabolism and thermogenesis [27]. At the 3 week time point, this protein was shown to be significantly dephosphorylated (−2.46 *p*-value 0.03). With the elimination of PGC-1 activity, the signal to initiate fatty acid catabolism would be lost. Both the loss of PGC-1 and the loss of inhibition ACC1 lead to increased fatty acid stores in the muscle. The above results can all proceed from a change in AMPK activity over time brought about by the *Salmonella* Typhimurium infection. The result of this infection-induced AMPK change may be a disruption of the normal metabolic functions within the muscle of the birds.

**Figure 4:**
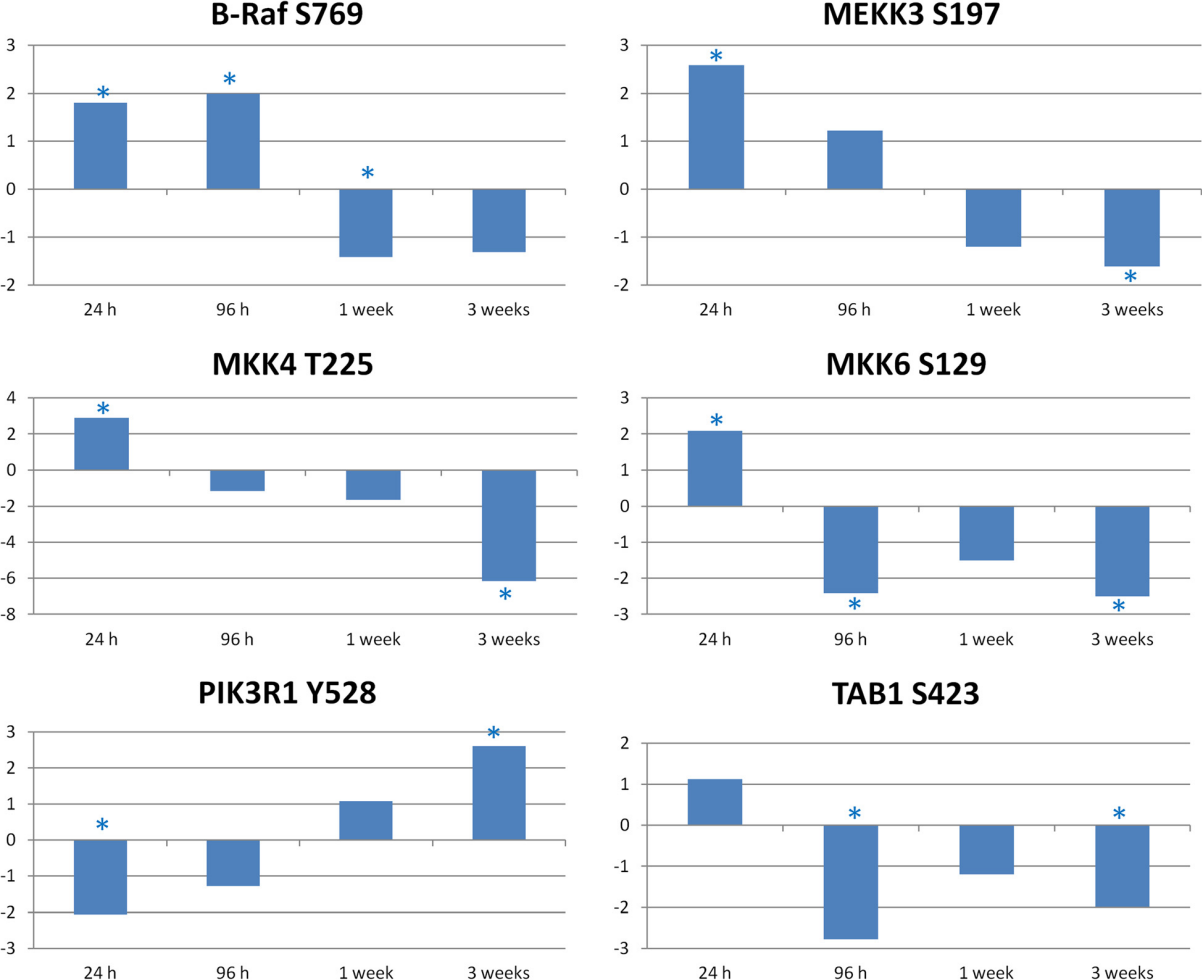
**Insulin/mTOR peptide changes relative to control over time.** Comparison of *Salmonella* Typhimurium infected to uninfected muscle samples at each time point generate a phosphoylation fold change and a statistical significance value for each peptide on the array. The * indicates statistically significant difference in phosphorylation relative to control (*p* < 0.05).

Based on the pathway and GO results described previously insulin signaling appeared to be affected by the infection. The KEGG insulin pathway showed a statistically significant change due to the infection at all time points (Table 1), and the GO Biological Process insulin receptor signaling pathway showed a statistically significant change at 24 h post infection (Table 2). In addition, the mTOR pathway showed statistically significant changes at three of the four time points. Due to the significant overlap between the two pathways, and the fact that many pathway intermediates appear in the peptide array analysis, we will consider a combined insulin/mTOR pathway in our analysis [15]. Several peptides representing protein phosphorylation sites known to be involved in the insulin/mTOR pathway showed significant change in phosphorylation state over multiple time points (Figure 4). The two branches of the insulin/mTOR signaling pathway are affected by the *Salmonella* Typhimurium infection over time: mitogen-activated protein kinase (MAPK) signaling and phosphoinositide 3-kinase (PI3K)/Akt (also known as Protein Kinase B) signaling. The majority of the peptides that had significant changes over multiple time points are part of the MAPK branch. Raf, mitogen-activated protein kinase kinase kinase 3 (MEKK3), mitogen-activated protein kinase kinase 4 (MKK4), mitogen-activated protein kinase kinase 6 (MKK6) and TAB1 are all part of MAPK-related signaling. In the PI3K branch at the 3 week time point, the phosphoinositide 3-kinase regulatory subunit 1 (PIK3R1) is activated by the phosphorylation ofY528 [28]. This phosphorylation indicates that at least part of the PI3K/Akt pathway is still active or is being activated by changes caused by the infection at the later time points.

**Figure 5:**
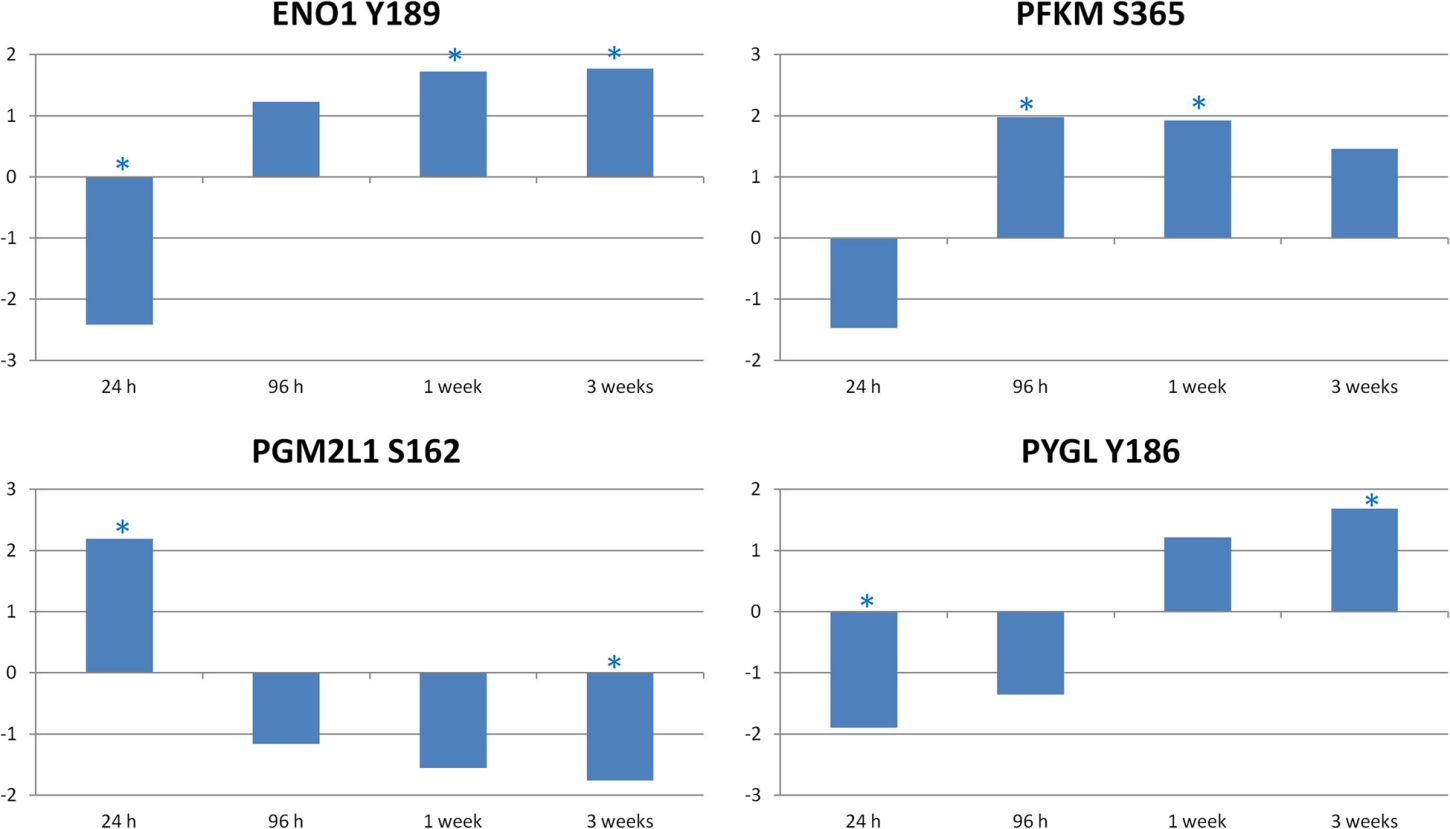
**Glucose related peptide changes relative to control over time.** Comparison of *Salmonella* Typhimurium infected to uninfected muscle samples at each time point generate a phosphoylation fold change and a statistical significance value for each peptide on the array. The * indicates statistically significant difference in phosphorylation relative to control (*p* < 0.05).

The function of insulin and its signaling pathway is intimately related to glucose metabolism. Indeed, one of the major functions of insulin is to stimulate the uptake of glucose by cells. Again considering the peptides that display significant differential phosphorylation at multiple time points, we see several peptides involved in glucose metabolism with a consistent change in response (Figure 5). Enolase 1 (ENO1), 6-phosphofructokinase, muscle type (PFKM) and glycogen phosphorylase (PYGL) all displayed increasing phosphorylation over the course of the experiment. Conversely, phosphoglucomutase 2-like 1 (PGM2L1) showed decreasing phosphorylation. PGM2L1 is an anabolic enzyme, and its decreased activation at the later time points while the other catabolic peptides were activated indicates an increase in the glucose-consuming response.

Overall, the results showed that the *Salmonella* Typhimurium infection has a number of effects on the insulin/mTOR pathway. Effects can be seen at various branch points including MAPK signaling, PI3K signaling and glucose related signaling. Considering the specific peptides described which showed differential phosphorylation over the course of the study, it is clear why insulin, mTOR, glycolysis/gluconeogesis and MAPK pathways were implicated by the KEGG pathway analysis generated by the STRING database (Table 1).

### Antibody peptide array: validation of kinome analysis

One of the standard methods for validating kinomic peptide array data is the use of phosphospecific antibodies. Normally, western blots using antibodies for specific phosphorylation events are performed to confirm the individual phosphorylation events reported by the array. This validation is akin to performing quantitative real-time polymerase chain reaction on individual genes to validate cDNA microarray data. We chose a slight variation of that standard validation processes. We employed an antibody microarray containing both pan-specific and phospho-specific antibodies. Despite the scarcity of chicken-specific antibodies, the key proteins of interest based on the peptide array results were relatively well conserved between humans and chickens, giving us confidence that we would observe significant cross-reactivity from the antibodies. The percent orthology between the human and chicken 15 amino acid phosphorylation target sites (as determined by NCBI Protein Blast analysis) is shown in Table 3. An antibody array covering the mTOR pathway was used. Comparing 1 week infected samples to 24 h infected samples allowed us to observe changes in the infected animal muscle at an early to late time course. Similarly, we were analyzing changes in phosphorylation over the early to late stages of infection with the peptide arrays. Following data normalization, the results pointed to a similar pattern to that observed with the peptide arrays (Table 3).

**Table 3:** Antibody array results

**Protein name (phosphorylated residue)**	**Fold change antibody array**	***P *-value**	**Orthology**	**Fold change peptide array**
4E-BP1 (Phospho-Ser65)	−1.43712	0.00137	93%	N/A
4E-BP1 (Phospho-Thr70)	−1.36207	0.013516	86%	N/A
AKT (Phospho-Ser473)	1.579649	0.002667	100%	1.61614
AKT (Phospho-Thr308)	1.898775	0.003797	100%	1.31019
AKT1 (Phospho-Ser124)	−1.47999	0.000402	100%	N/A
AKT1 (Phospho-Thr450)	−1.5584	0.000476	100%	N/A
AKT1S1 (Phospho-Thr246)	−1.79817	2.22E-05	0%	N/A
AMPK1 (Phospho-Thr174)	−1.22623	0.0154	100%	−1.09134
AMPKbeta1 (Phospho-Ser182)	−1.34017	0.029026	93%	−1.10925
BAD (Phospho-Ser112)	1.40376	0.012781	78%	N/A
BAD (Phospho-Ser134)	1.380989	0.020394	0%	N/A
BAD (Phospho-Ser136)	1.29749	0.039652	0%	N/A
eIF4E (Phospho-Ser209)	−1.33384	0.030631	100%	N/A
ERK3 (Phospho-Ser189)	1.210088	0.019255	100%	4.01876
GSK3 beta (Phospho-Ser9)	2.974451	0.002007	100%	1.12689
IR (Phospho-Tyr1355)	3.187483	2.62E-05	53%	N/A
IR (Phospho-Tyr1361)	1.685564	0.012336	93%	N/A
P70S6K (Phospho-Ser371)	−1.73418	0.000111	100%	N/A
P70S6K (Phospho-Ser418)	−1.58607	0.000109	100%	N/A
P70S6K (Phospho-Ser424)	−1.39268	0.037721	100%	N/A
P70S6K (Phospho-Thr229)	−1.44379	0.000416	100%	N/A
P70S6K (Phospho-Thr389)	−1.84347	0.009943	100%	−1.12207
P70S6K (Phospho-Thr421)	−1.22221	0.013127	100%	N/A
P70S6k-beta (Phospho-Ser423)	1.60054	0.000107	100%	N/A
P90RSK (Phospho-Ser380)	1.255345	0.04783	100%	−1.12470
PDK1 (Phospho-Ser241)	1.632697	0.002201	93%	−1.01557
PI3-kinase p85-alpha (Phospho-Tyr607)	−1.3092	0.038374	87%	−1.34043
PIP5K (Phospho-Ser307)	−1.2407	0.026195	100%	N/A
PTEN (Phospho-Ser370)	1.491624	0.002252	100%	N/A
PTEN (Phospho-Ser380)	1.533421	0.005982	100%	1.12240

Statistically significant (*p* < 0.05) phosphospecific antibody array analysis results of *Salmonella* Typhimurium infected muscle samples. One week post-infection samples were compared to 24 h post-infection samples to find changes in infected muscle over time. Antibodies due to being bound to phosphorylated protein had a statistically significant difference in fluorescent signal are shown. Fold Change Antibody Array is the change in fluorescent signal when comparing the 1 week and 24 h infected samples as indicated by the antibody array. Orthology indicates the % similarity between human and chicken at the 15 amino acid region flanking the phosphorylation residue. Fold Change Peptide Array is the change in fluorescent signal when comparing the 1 week and 24 h infected samples as indicated by the peptide array. N/A indicates the exact phosphorylation target residue on the antibody array was not present on the peptide array.

Analysis of the antibody array data via the STRING data based showed that the top two KEGG pathways were insulin signaling pathway (*p* value 9.50 × 10^-10^) and mTOR signaling pathway (*p* value 1.08 × 10^-7^). GO biological processes indicated insulin receptor signaling pathway (*p* value 2.98 × 10^-6^), and GO molecular function showed ATP binding (*p* value 1.14 × 10^-3^). All of these results were in agreement with the peptide array data. In addition, specific results such as the dephosphorylation of AMPK, activation of insulin receptor, activation of Akt (S374) and shutting down of the mTOR pathway (decreases in P70S6K, 4E-BP1 and eIF4E) are in agreement with peptide array results.

## Discussion

Peptide array kinome analysis has been used for the study of various cell biology questions [5,6]. In addition, species-specific peptide arrays have been employed to perform this type of high-throughput analysis on species other than humans or mice [8,9]. This technique has uncovered novel biology and advanced our understanding of a number of biological processes and disease states [8-10]. In this study, we have used the species-specific peptide arrays to uncover metabolic signaling changes in chicken skeletal muscle due to *Salmonella* Typhimurium infection. To our knowledge, this study incorporates a number of firsts in the field of kinomics: the first published use of a chicken-specific peptide array, the first study of metabolic processes using a kinome array and the first study of systemic phosphorylation-mediated metabolic changes brought about by a gastrointestinal infection.

The results of this study clearly point to changes in key, related, metabolic processes upon *Salmonella* Typhimurium infection: insulin/mTOR/glucose, fatty acid and AMPK related signaling. Metabolic changes in peripheral tissue as a result of infection have been reported previously [29]; however, metabolic changes as a result of a sub-clinical infection, especially one that is considered well tolerated such as a *Salmonella* Typhimurium infection in chicken, has not previously been shown. To our knowledge, this is the first study demonstrating that *Salmonella* Typhimurium infection of the chicken cecum has potential systemic effects on metabolism/physiology. The consequences of this may be a direct effect on animal performance, including muscle quality, immunity and overall health.

**Figure 6:**
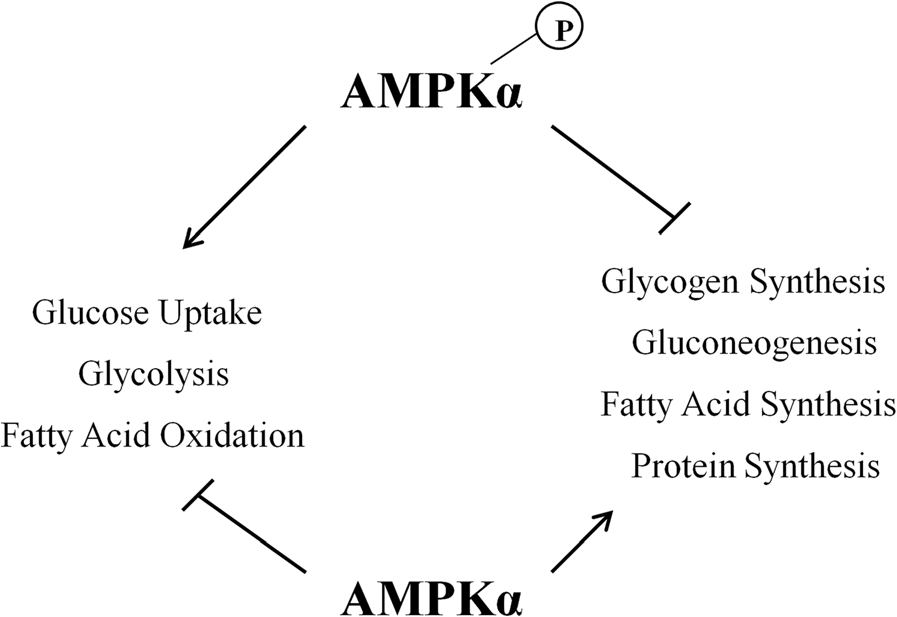
**AMPK energy influence.** The phosphorylated, active form of AMPK exerts effects that lead to an increase in ATP-producing functions and a decrease in ATP-consuming functions. When AMPK is not phosphorylated, these signals are not present, rather, the ATP producing signals are now present, and the ATP consuming functions are able to occur.

**Figure 7:**
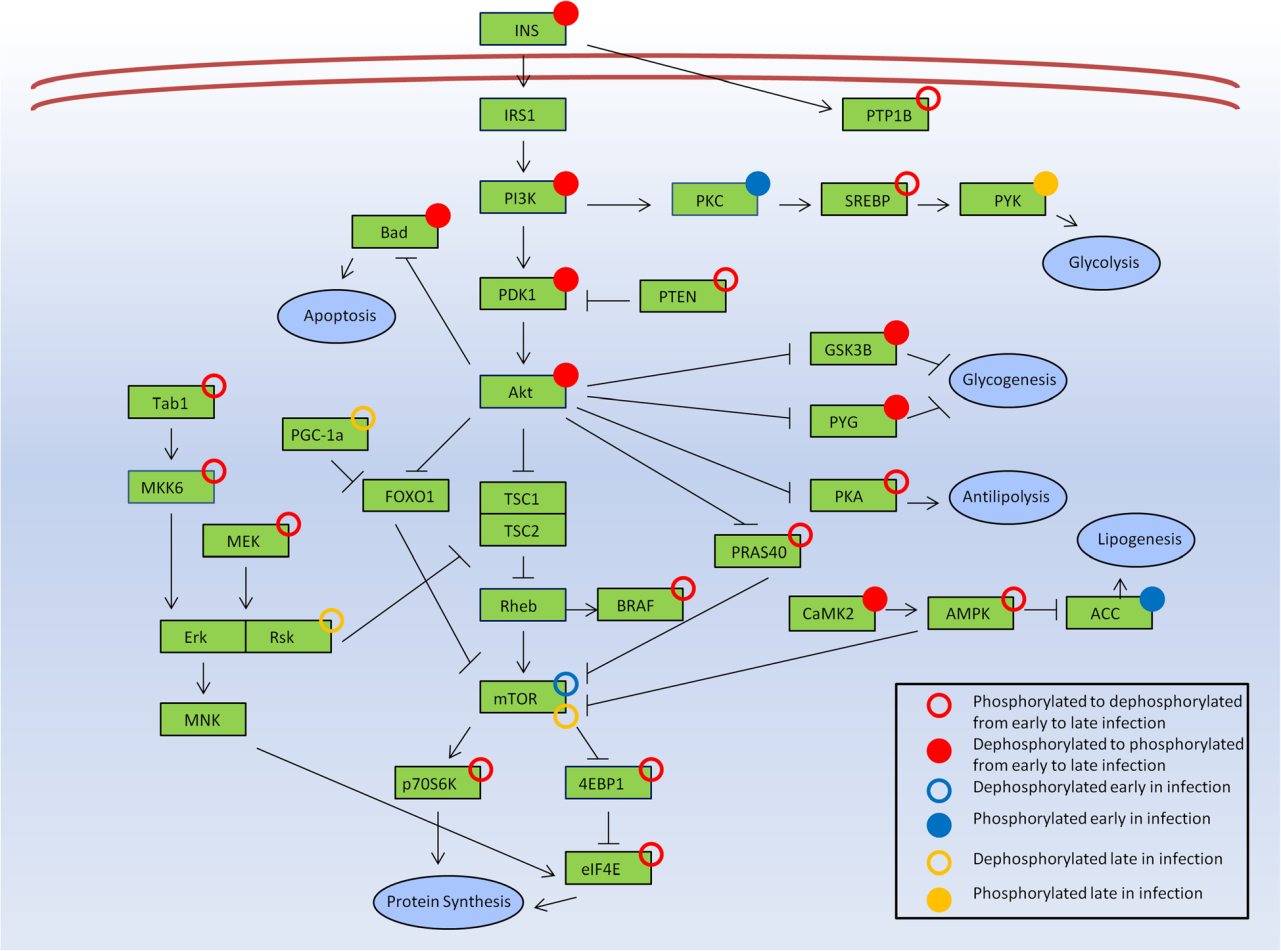
**Pathway summary of peptide array and antibody array results.** Figure displays an overview of the observed results combining both peptide array and antibody array techniques. Phosphorylation/dephosphorylation refers to phosphorylation state of peptides comparing infected to uninfected samples (peptide arrays) or late to early infected samples (antibody arrays). Early changes refer to differential phosphorylation at the 24 h and/or 96 h time points. Late changes refer to differential phosphorylation at the 1 week and/or 3 week time points.

AMPK is a key metabolic regulator. Through AMP binding domains and phosphorylation sites for a number of metabolic related kinases, AMPK can receive signals of cellular energy state and then send corresponding signals via phosphorylation [13]. These signals can affect cellular processes such as glycolysis/gluconeogenesis, protein synthesis, fatty acid synthesis and fatty acid oxidation, among others (Figure 6). Clear changes in AMPK phosphorylation state were observed at various time points following infection with *Salmonella* Typhimurium. Phosphorylation sites on all three of the AMPK subunits displayed differential phosphorylation based on the peptide array data, and both alpha and beta subunits showed significant change between the 24 h and 1 week post infection time points on the antibody arrays. The decreased phosphorylation of AMPKα, which is shown by both the peptide array and antibody array techniques, indicates that AMPK activity is decreasing over time. Active AMPK, seen at the early time points, would lead to the activation of catabolic, ATP producing functions such as glycolysis and fatty acid oxidation. At the later time points where a decreased phosphorylation of AMPKα is observed, the effect would be a decrease in ATP producing functions and an increase in anabolic, ATP consuming activities such as lipogenesis. Figure 7 shows the pathways that lead to an early inhibition of lipogenesis that is lost over time. The profound effects that AMPK has on lipid metabolism and whether chicken muscle is lean or fat has been reported previously [23]. A loss of AMPK related inhibition of lipogenesis could result in increased fat deposition in the muscle of infected birds. This ATP energy consumption could be directed toward other healthier purposes such as immune response or building muscle. As can be observed in Figure 7, the mTOR pathway leading to protein synthesis was inactivated at the later time points of infection. It is possible that energy is being taken from a peptide synthesis function and consumed for a disregulated Lipogenesis function.

The decreased phosphorylation of AMPKα would normally lead to an increase in gluconeogenesis (an ATP consuming function) and a decrease in glycolysis. As can be observed in the phosphorylation states of several glucose related proteins (Figure 5) and the insulin/mTOR pathway (Figure 7), glycolysis actually increases over time, and there is an active inhibition of gluconeogensis as evidenced by the phoshorylation of GSK3β and PYG. As the protein synthesis pathway appears to be inactive, it is unlikely that the energy being mobilized by glycolysis is being used to synthesize protein and build muscle. It is possible that this glucose mobilization is a response to a lack of energy due to the pro-anabolic, ATP consuming response initiated by the inhibition of AMPK. An example of this type of response may be lipogenesis activation. Regardless of where this energy is being used, the mobilization of glucose stores and the lack of AMPK signaling indicate a serious disruption of the normal metabolic functions within the muscles of *Salmonella* Typhimurium infected birds. We can see that protein synthesis, one of the normal functions of a rapidly growing animal, is deactivated (Figure 7). The number of links between infection, pathogenesis, immunity and metabolism are so numerous that a consideration of metabolism when studying host-pathogen interaction is important. Taken together, these results indicate that though *Salmonella* Typhimurium has been considered a non-disease causing agent in chickens, it does result in serious disruption of metabolic functions with potential consequences for the normal physiological function and health of the animal.

Results from the antibody microarray provide a further characterization of *Salmonella* induced muscle metabolism changes. The peptide array data at the later time points showed that the PI3K/Akt pathway appears active from the level of the receptor to Akt; however, mTOR and proteins downstream appear inactive. The phosphorylation of proline-rich AKT1 substrate 1 (PRAS40) (also named AKT1S1) by Akt2 at T246, shown on the antibody array, provides further evidence for active insulin related signaling. PRAS40/AKT1S1 is an inhibitor of mTOR, but phosphorylation at T246 inhibits PRAS40/AKT1S1 inhibition of mTOR [30]. As indicated by the peptide array Akt2 is phosphorylated at a site that would indicate increased enzymatic activity. Akt2 then phosphorylates PRAS40 at T246, as shown by the antibody array, this phosphorylation inhibits PRAS40 activity. Active PRAS40 inhibits mTOR signaling while inhibited PRAS40 does not. Despite PRAS40 inhibition we observe a dephosphorylation and deactivation of the mTOR signaling pathway starting at the mTOR peptide itself. A possible explanation for the shutting down of mTOR activity, despite PRAS40 inhibition, is by an alternative Akt-independent method, through phosphatase and tensin homolog (PTEN) activity. The antibody microarray shows that PTEN is phosphorylated at S370 and S380, while the current belief is that these phosphorylations inhibit some PTEN functions, there is as yet no consensus on how these phosphorylation events affect enzymatic activity specifically [31]. These data highlight the importance of understanding the results of individual phosphorylation events on a given protein. An increased phosphorylation does not always indicate activation or a dephosphorylation inactivation. Between the peptide array and antibody microarray, we were able to achieve good coverage of the insulin/mTOR signaling pathway; however, numerous other proteins, which were not included in either the peptide array or antibody array, may be exerting their effects as well. For example, mTOR shutdown may be a result of forkhead box protein O1 (FOXO1) activity. FOXO1 activity was also shown to decrease the phosphorylation and activity of not only mTOR but also p70S6K, 4EBP1 and eIF4E [32], all results observed in the antibody microarray. At this later time of infection it is also possible that effector molecules produced by *Salmonella* Typhimurium have gone systemic and are altering metabolic signaling in the peripheral muscle.

It had been assumed that an infection with *Salmonella*, though dangerous from a food safety perspective, was not a detriment to the health and growth of commercial chickens [3]. However, this study shows that effects from infection can be observed in skeletal muscle metabolism. The range of ways that an infection may be altering host metabolism is of such wide scope that substantial follow-up studies will be required to uncover the potential mechanism(s). There are a number of ways that a *Salmonella* Typhimurium infection may alter muscle metabolism:

1. A transient response to early infection may activate stress responses, including the mobilization of energy by AMPK. Following the establishment of infection, either the stress response may cease or AMPK may become desensitized to the signal resulting in the inverse response of fatty acid synthesis. This desensitization of AMPK has been observed previously in response to repeated exercise [33].

2. The normal gut microbiota provides a large, complex assortment of signals, metabolites and stimulations to the host. An infection of *Salmonella* would almost certainly disrupt the normal population balance of the gut and alter these signals [34]. Ample evidence is emerging as to the importance of the gut microbiota and the balance of microbiotic populations for animal health [35]. A persistent *Salmonella* infection of the chicken gut must be exerting effects on the other bacteria inhabiting the system. *Salmonella* infection has been shown previously to cause changes in the microbial ecology of the gastrointestinal tract of mice [36]. A previous study has shown that an antibiotic-induced disruption of the gut microbiota alters host susceptibility to enteric infection [37]. Even though *Salmonella* infection does not result in obvious negative consequences to an avian host, it may disrupt natural beneficial bacteria. It is possible that these are the same or similar beneficial bacteria that thrive following animal treatment with growth enhancing antibiotics. Following antibiotic treatment of food animals, gut microbiota populations are disrupted and those bacteria beneficial to growth are able to thrive [38]. In our study, the opposite may be occurring, with *Salmonella* out-competing the beneficial bacteria. The infection at later time points causes an increase in fatty acid synthesis, a negative consequence in terms of meat-animal production. For many years, there has been evidence of an effect on chicken adiposity due to infections [39].

3. Another possible cause of systemic changes in the host is changes in hormones and/or hormone metabolism. *Salmonella* Typhimurium infection has been shown to cause large scale changes in host hormone metabolism [40]. Metabolomic study of the liver and feces of mice infected with *Salmonella* Typhimurium showed changes in a variety of metabolites including those involved in fatty acid and glucose metabolism. In addition, it was found that significant changes in eicosanoid metabolism intermediates occurred due to *Salmonella* Typhimurium. Eicosanoids are fatty acid derived hormones which can have systemic effects. It is possible that the muscle metabolism changes we have observed here are a result of changes in hormones, specifically eicosanoids, brought about by the intestinal infection of the host. cPLA2 phosphorylation is evidence of eicosanoid hormone influence. cPLA2 is an enzyme that catalyzes an early step in the biosynthesis of eiconsaoid hormones including the prostaglandins [41]. We see a significant increase in cPLA2 phosphorylation over the course of this study (Figure 3), that may result in an increase in hormone production. These hormones may have systemic effects resulting in the changes in muscle metabolism we have observed.

4. There are numerous nervous system connections between the brain and gut. These connections can influence metabolism throughout the body, including glucose metabolism [42]. If a *Salmonella* infection, either through altered gut microbiota populations or altered gut absorption, alters these neuronal signals, it could affect metabolism in the entire body. A lack of nutrient absorption could signal the body to engage energy stores in the muscle, adipose cells or the liver, for example. Any or all of the potential *Salmonella* effects on the gut described above could affect skeletal muscle metabolism in the ways observed in this study.

In summary, this study shows that skeletal muscle metabolism is altered following *Salmonella* Typhimurium infection, and that these alterations change over time. Early activation of AMPK indicates an energy-producing catabolic response, while late AMPK deactivation would lead to an anabolic response which appears biased toward fatty acid metabolism. The disregulation of the mTOR/insulin signaling pathway points to altered glucose metabolism throughout the time course of the study, with a strong inhibition of glycogenesis at the later time points. This study also adds to the growing body of evidence pointing to the importance of gut bacterial populations in systemic host functions.

## Abbreviations

Salmonella Typhimurium: *Salmonella enterica* serovar Typhimurium; AMPK: 5'-adenosine monophosphate-activated protein kinase; mTOR: mammalian Target of rapamycin; ACUC: Animal Care and Use Committee; GO: Geneontology; KEGG: Kyoto Encyclopedia of Genes and Genomes; STRING: Search Tool for the Retrieval of Interacting Genes; FDR: False discovery rate; CaMK2: Calcium/calmodulin-dependent protein kinase II; EF2K: Eukaryotic elongation factor-2 kinase; cPLA2: cytosolic phospholipase A2; CPT1A: carnitine Palmitoyltransferase 1A; ACC1: Acetyl-coenzyme A carboxylase 1; PGC-1: PPAR gamma co-factor 1; MAPK: Mitogen-activated protein kinase; PI3K: Phosphoinositide 3-kinase/Akt; MEKK3: Mitogen-activated protein kinase kinase kinase 3; MKK4: Mitogen-activated protein kinase kinase 4; MKK6: Mitogen-activated protein kinase kinase 6; PIK3R1: Phosphoinositide 3-kinase regulatory subunit 1; ENO1: Enolase 1; PFKM: 6-phosphofructokinase, muscle type; PYGL: glycogen phosphorylase; PGM2L1: Phosphoglucomutase 2-like 1; PRAS40 or AKT1S1: AKT1 substrate 1; PTEN: Phosphatase and tensin homolog; FOXO1: Forkhead box protein O1.

## Competing interests

The authors declare that they have no competing interests.

## Authors’ contributions

MK and RA conceived the experiment and wrote the manuscript. RA carried out the experiment and analyzed the data. SN designed the peptide array. All authors read and approved the final manuscript.

## Supplementary Material

Additional file 1 **Peptide array results.** Complete peptide array results for all 300 peptides on the array and all 4 time points. Results represent the average of 3 separate animals combined to produce a representative result. Protein and Target Amino Acid show the protein name represented by the peptide and phosphorylated amino acid residue printed onto the array. Human Accession number indicates the human equivalent protein. Fold change is the relative phosphorylation compared to the time-matched control. *P*-value is the statistical significance of the differential phosphorylation. Click here for file
