# Supplementary material for: Salmonella enterica Typhimurium infection causes metabolic changes in chicken muscle involving AMPK, fatty acid and insulin/mTOR signaling
Source: Vet Res. 2013 May 17;44(1):35. doi: 10.1186/1297-9716-44-35 (PMC3663815; doi:10.1186/1297-9716-44-35)
Supplement: Additional file 1 — Peptide array results. Complete peptide array results for all 300 peptides on the array and all 4 time points. Results represent the average of 3 separate animals combined to produce a representative result. Protein and Target Amino Acid show the protein name represented by the peptide and phosphorylated amino acid residue printed onto the array. Human Accession number indicates the human equivalent protein. Fold change is the relative phosphorylation compared to the time-matched control. P-value is the statistical significance of the differential phosphorylation. [file 1297-9716-44-35-S1.docx]

**Additional File 1 Peptide array results.**

|  |  | **24 h** | | **96 h** | | **1 week** | | **3 weeks** | |
| --- | --- | --- | --- | --- | --- | --- | --- | --- | --- |
| **Protein and**  **Target Amino Acid** | **Human Accession** | **Fold Change** | ***P* -value** | **Fold Change** | ***P* -value** | **Fold Change** | **P -value** | **Fold Change** | ***P* -value** |
| ABCC1 Y489 | P33527 | 2.227967 | 0.010455 | -1.18773 | 0.336376 | 1.290444 | 0.311903 | 1.841585 | 0.145029 |
| ACC1 S1255 | Q13085 | 2.181159 | 0.042942 | -1.68473 | 0.114966 | 1.190842 | 0.335469 | 1.738345 | 0.129459 |
| AKt1 S473 | P31749 | -1.69329 | 0.13878 | -1.30043 | 0.237038 | 1.23099 | 0.155795 | 2.178441 | 0.085926 |
| AKt1 T308 | P31749 | -1.64864 | 0.135052 | -2.30688 | 0.020268 | -1.17484 | 0.218558 | 1.368722 | 0.320776 |
| AKt2 S472 | P31751 | -1.30686 | 0.157011 | -1.20942 | 0.236655 | -1.38345 | 0.267116 | 1.541497 | 0.03679 |
| AKt2 T308 | P31751 | -1.87992 | 0.003592 | -1.51262 | 0.103256 | 1.406235 | 0.112309 | 1.687422 | 0.076268 |
| ALDOA Y204 | P04075 | 2.279448 | 0.018195 | 1.095191 | 0.407305 | 2.197696 | 0.023351 | 1.802048 | 0.046361 |
| ALDOA Y223 | P04075 | -2.02529 | 0.018278 | 1.691662 | 0.129838 | -1.02721 | 0.47806 | 1.727376 | 0.065636 |
| ALDOB T39 | P05062 | 1.95847 | 0.110482 | -1.78399 | 0.14586 | -1.29925 | 0.157744 | -1.41978 | 0.263623 |
| ALKBH5 T294 | Q6P6C2 | 1.56093 | 0.105852 | 1.294442 | 0.135405 | 1.281442 | 0.171014 | 1.08819 | 0.397585 |
| AMPKA1 S497 | Q13131 | 1.805326 | 0.055738 | 1.346305 | 0.142818 | 1.374807 | 0.188196 | -1.68658 | 0.187648 |
| AMPKA1 T173 | Q13131 | -1.16509 | 0.352698 | 1.474074 | 0.169833 | 1.11263 | 0.397141 | 1.09476 | 0.238828 |
| AMPKA1 T184 | Q13131 | -1.21401 | 0.366377 | -1.26251 | 0.324153 | -1.52961 | 0.092681 | -2.6591 | 0.028626 |
| AMPKA2 S377 | P54646 | 3.47664 | 0.022073 | -1.21725 | 0.352507 | -1.74609 | 0.139118 | -2.65771 | 0.000229 |
| AMPKA2 S377 | P54646 | 3.523163 | 0.007061 | 1.684401 | 0.049862 | -1.37928 | 0.197651 | -2.32791 | 0.041694 |
| AMPKB1 S111 | Q9Y478 | -1.14951 | 0.382902 | 1.334192 | 0.08912 | -1.23217 | 0.288273 | 1.896792 | 0.166227 |
| AMPKB1 S184 | Q9Y478 | -1.20008 | 0.351056 | 1.298515 | 0.246529 | -1.59176 | 0.235912 | -1.66129 | 0.147111 |
| AMPKB2 S110 | O43741 | -2.03332 | 0.013582 | 1.282051 | 0.286541 | 1.411949 | 0.25103 | 3.307116 | 0.005065 |
| AMPKB2 S186 | O43741 | -1.33772 | 0.297462 | -1.60838 | 0.064972 | -1.25599 | 0.222004 | -1.6099 | 0.112453 |
| AMPKG2 T160 | Q9UGJ0 | 1.116149 | 0.362332 | -1.36195 | 0.200221 | -1.15109 | 0.338546 | -2.55536 | 0.000437 |
| AMPKG3 S148 | Q9UGI9 | -1.93834 | 0.01673 | -1.39833 | 0.264014 | 2.684927 | 0.020477 | 3.38516 | 0.009595 |
| AP-2 beta S247 | Q92481 | 1.3646 | 0.303527 | -1.687 | 0.134558 | -1.26681 | 0.098867 | -2.03448 | 0.073162 |
| AP-2 mu2 T154 | Q96CW1 | -1.02014 | 0.456051 | -1.26997 | 0.186327 | 1.011728 | 0.484597 | 1.775816 | 0.087355 |
| A-Raf T260 | P10398 | 1.200923 | 0.232832 | -1.37484 | 0.119149 | -1.00113 | 0.498936 | -2.02385 | 0.181684 |
| ARHGAP17 S485 | Q68EM7 | 1.769275 | 0.086174 | 1.045293 | 0.421834 | 1.644188 | 0.131976 | -1.01399 | 0.48385 |
| ARHGAP6 Y391 | O43182 | -1.62837 | 0.16151 | 1.473638 | 0.075221 | -1.00291 | 0.497811 | 1.250812 | 0.348351 |
| ASK1 S852 | Q59GL6 | -1.29453 | 0.164458 | 1.136488 | 0.31078 | -1.12834 | 0.397604 | -1.14686 | 0.367611 |
| ASK1 T724 | Q59GL6 | -1.35604 | 0.165086 | -1.43651 | 0.098688 | -1.67013 | 0.04727 | 1.61103 | 0.145718 |
| ATP12A T356 | P54707 | -1.18853 | 0.320494 | -1.03334 | 0.460654 | 1.015584 | 0.481561 | -1.12645 | 0.373956 |
| ATP5B T317 | P06576 | -1.27095 | 0.108348 | 1.024156 | 0.451182 | 1.02107 | 0.466716 | -1.79696 | 0.070097 |
| BCAP S639 | Q6ZUJ8 | -1.65022 | 0.172362 | -1.13421 | 0.336741 | 1.007366 | 0.488757 | 1.705972 | 0.043962 |
| BLNK Y91 | Q8WV28 | 1.450972 | 0.195362 | -1.14102 | 0.376635 | -1.42398 | 0.161535 | -1.45674 | 0.180754 |
| B-Raf S486 | P15056 | -1.50185 | 0.085946 | 1.058091 | 0.420112 | -1.64377 | 0.063808 | -1.54569 | 0.13294 |
| B-Raf S769 | P15056 | 1.805991 | 0.044728 | 1.997651 | 0.034648 | -1.41715 | 0.017119 | -1.31617 | 0.274699 |
| caldesmon S672 | Q05682 | 1.722972 | 0.128131 | 1.197875 | 0.303244 | -1.03132 | 0.476963 | 1.380716 | 0.129397 |
| caldesmon S702 | Q05682 | -2.38777 | 0.005752 | 1.418636 | 0.128587 | 1.587685 | 0.136047 | 1.345385 | 0.2165 |
| CALM2 Y100 | NP 001734 | -1.00749 | 0.489909 | 1.251094 | 0.151487 | 1.406392 | 0.164316 | -1.47345 | 0.166275 |
| calmodulin S82 | P62158 | 1.817382 | 0.162548 | -1.55322 | 0.104142 | -2.13954 | 0.006459 | -1.4765 | 0.190529 |
| Calpain-2 T370 | P17655 | 1.799213 | 0.046737 | -1.26614 | 0.283173 | 1.340125 | 0.282181 | -1.10397 | 0.429795 |
| Calponin 1 Y261 | P51911 | -1.53991 | 0.199336 | -1.13969 | 0.362863 | 1.503215 | 0.097845 | 1.462282 | 0.10457 |
| calponin 2 Y184 | Q99439 | -1.91324 | 0.07126 | -1.27151 | 0.148567 | 2.411824 | 0.009481 | 1.344823 | 0.221593 |
| Calponin 3 S293 | Q15417 | -1.09239 | 0.368617 | -1.22028 | 0.256035 | -1.16724 | 0.192381 | -1.3546 | 0.101764 |
| CaMK2-alpha T532 | Q9UQM7 | 1.365888 | 0.143463 | -1.59769 | 0.159871 | 1.455205 | 0.128536 | 1.239981 | 0.31103 |
| CaMK2-beta T287 | Q13554 | 2.732632 | 0.00556 | -2.08043 | 0.07605 | 1.362956 | 0.079302 | -1.16548 | 0.33849 |
| CaMK2-delta T336 | Q13557 | -1.06706 | 0.436819 | -1.42626 | 0.123102 | -2.20043 | 0.007147 | -1.21074 | 0.367387 |
| CaMK2-gamma T287 | Q13555 | -1.65417 | 0.037284 | -1.78708 | 0.077422 | 1.100531 | 0.43273 | -1.61739 | 0.05262 |
| CAMKK1 S106 | Q8N5S9 | 1.481992 | 0.107307 | -1.24007 | 0.194425 | 1.042547 | 0.444978 | 1.216446 | 0.146812 |
| CAMKK1 S507 | Q8N5S9 | -1.2792 | 0.16531 | -1.11227 | 0.374438 | 1.474469 | 0.167902 | -1.43015 | 0.132986 |
| CAMKK2 S515 | Q96RR4 | -1.24562 | 0.221714 | -1.03886 | 0.459463 | 1.754161 | 0.020185 | -1.66896 | 0.091587 |
| CAPN7 Y620 | Q9Y6W3 | -1.16068 | 0.37165 | 1.096038 | 0.372513 | -1.14145 | 0.328636 | -1.47522 | 0.07338 |
| CAPNS1 S34 | P04632 | 2.227658 | 0.032048 | -1.39816 | 0.162135 | -1.34109 | 0.220872 | -1.11847 | 0.301055 |
| CARD11 S118 | Q9BXL7 | -3.99928 | 0.000793 | -1.04691 | 0.455738 | 1.222645 | 0.152373 | 1.530899 | 0.152566 |
| Cdc37 S13 | Q16543 | 1.433523 | 0.220887 | 1.467313 | 0.13374 | 1.117088 | 0.40709 | 3.850696 | 0.006522 |
| CDK1 T161 | P06493 | -1.82282 | 0.036404 | 1.231862 | 0.163821 | -1.29579 | 0.257757 | 1.012722 | 0.48551 |
| CDK1 Y15 | P06493 | -1.88076 | 0.038327 | 1.163737 | 0.382815 | 1.503294 | 0.054973 | 1.064302 | 0.371237 |
| CDK2 T160 | P24941 | -1.17927 | 0.362244 | 1.121159 | 0.259905 | 1.259978 | 0.326992 | 2.266351 | 0.033544 |
| CDK2 Y15 | P24941 | -3.19447 | 0.001666 | -1.05668 | 0.33381 | 1.34296 | 0.118101 | 2.060906 | 0.153064 |
| CDK3 Y15 | Q00526 | -1.91087 | 0.011786 | -1.13905 | 0.272185 | 1.322973 | 0.21 | 1.86106 | 0.066217 |
| CDK5 S159 | Q00535 | -1.79478 | 0.00259 | 1.142549 | 0.188252 | 1.533691 | 0.05875 | 4.020098 | 0.002761 |
| CDK6 T177 | Q00534 | -1.26292 | 0.139108 | -1.10595 | 0.287383 | 1.402957 | 0.140392 | 1.264824 | 0.3293 |
| CDK6 Y24 | Q00534 | -1.5205 | 0.049977 | -1.11217 | 0.325275 | 1.700871 | 0.060352 | -1.64212 | 0.042411 |
| CDK7 T142 | P50613 | -1.2373 | 0.213762 | 1.979633 | 0.014259 | -1.11575 | 0.390455 | 3.317695 | 0.022114 |
| CDK9 S175 | P50750 | -1.60243 | 0.132494 | -1.13989 | 0.249724 | 1.450387 | 0.117533 | -1.56199 | 0.061159 |
| CDK9 T186 | P50750 | -1.43639 | 0.04232 | 1.466506 | 0.037984 | -1.12904 | 0.373632 | 1.881885 | 0.082572 |
| CDKAL1 Y146 | Q5VV42 | 1.124718 | 0.365731 | -1.50671 | 0.15706 | 1.139535 | 0.376906 | 1.211025 | 0.167638 |
| CHIP S284 | Q9UNE7 | 1.065387 | 0.444331 | -1.37538 | 0.219564 | -1.40522 | 0.187006 | 1.324232 | 0.277042 |
| Chk2 T364 | O96017 | 1.597579 | 0.115241 | 1.295948 | 0.237863 | 1.286693 | 0.276182 | -1.13792 | 0.14944 |
| CK1-A S242 | P48729 | -1.17052 | 0.331786 | 1.134437 | 0.364897 | 1.186607 | 0.245227 | -1.18061 | 0.181927 |
| CK1-D S430 | P48730 | 2.110368 | 0.005613 | 1.215641 | 0.176665 | 1.310215 | 0.220619 | -1.66741 | 0.167992 |
| CK1-E T44 | P49674 | -1.02543 | 0.465433 | -1.07186 | 0.436649 | 1.320402 | 0.280138 | 1.875476 | 0.052628 |
| CK1-G1 Y262 | Q9HCP0 | -1.87107 | 0.035119 | 1.027606 | 0.467896 | 1.258288 | 0.179895 | 1.175719 | 0.251743 |
| CK2-A1 T360 | P68400 | -1.21412 | 0.254119 | 2.120137 | 0.006378 | -1.12183 | 0.312176 | 1.428619 | 0.230528 |
| CK2-A2 S18 | P19784 | -1.60606 | 0.031654 | 1.171395 | 0.404325 | 1.937327 | 0.090096 | -1.03817 | 0.463792 |
| CK2-A2 Y256 | P19784 | -2.10188 | 0.036491 | 1.163653 | 0.283184 | 2.699906 | 0.021007 | -1.07706 | 0.407634 |
| CK2-B S209 | Q5SRQ6 | -1.3744 | 0.297322 | 1.60044 | 0.07812 | -1.80142 | 0.053117 | 2.567925 | 0.038006 |
| CKS1 Y8 | P61024 | -1.05432 | 0.443036 | 1.020837 | 0.461481 | -1.2613 | 0.179327 | 2.488849 | 0.048032 |
| CKS2 Y17 | P33552 | -2.64803 | 0.005299 | 1.00692 | 0.487955 | 1.734558 | 0.05809 | -1.0035 | 0.496653 |
| claudin-12 S229 | P56749 | -1.39606 | 0.239374 | -1.64907 | 0.102648 | 1.547545 | 0.050687 | 1.632904 | 0.166743 |
| CMPK S70 | P30085 | -1.17754 | 0.250211 | 1.247125 | 0.261159 | 1.37289 | 0.173687 | 3.764812 | 0.006226 |
| cPLA2 S228 | P47712 | -1.96351 | 0.046319 | 1.49268 | 0.026975 | 1.670091 | 0.073644 | 4.21505 | 0.000458 |
| CPT1A Y165 | P50416 | -1.0032 | 0.497058 | 1.849273 | 0.022988 | 1.79102 | 0.011806 | 1.169697 | 0.400003 |
| CRF-R1 S306 | P34998 | 1.708233 | 0.035296 | -1.35548 | 0.162042 | 1.02439 | 0.440025 | -2.78542 | 0.026675 |
| CrK Y222 | P46108 | 1.135391 | 0.33719 | -1.58404 | 0.200821 | 1.040691 | 0.453786 | -1.41381 | 0.238062 |
| CrK Y240 | P46108 | 1.147958 | 0.340823 | -1.62172 | 0.045988 | -1.06115 | 0.394755 | 2.517553 | 0.019674 |
| CRK7 Y892 | Q9NYV4 | 1.510228 | 0.196785 | -1.40434 | 0.219817 | 1.30024 | 0.276925 | 1.350209 | 0.198359 |
| CrkL Y207 | P46109 | 1.752795 | 0.140422 | -1.02009 | 0.473546 | 1.205837 | 0.284524 | -1.1028 | 0.250495 |
| CRMP-2 S522 | Q16555 | 1.900462 | 0.06642 | 1.190287 | 0.299893 | 1.686824 | 0.051095 | -1.2787 | 0.204407 |
| CRMP-4 T507 | Q6DEN2 | 2.574028 | 0.031708 | 1.082902 | 0.403985 | 1.270342 | 0.29268 | 1.074971 | 0.408781 |
| CTCF S604 | P49711 | 3.298564 | 0.003711 | -1.35974 | 0.216653 | -1.17943 | 0.237041 | 1.732053 | 0.071027 |
| DAB1 Y220 | O75553 | 1.729288 | 0.075623 | 1.141753 | 0.331999 | -1.08912 | 0.42902 | -1.70844 | 0.066443 |
| DAB1 Y232 | O75553 | 1.776981 | 0.053138 | -1.80362 | 0.054536 | 1.021221 | 0.464737 | 1.290995 | 0.308953 |
| DAGLBETA S424 | Q8NCG7 | 1.23645 | 0.140914 | -1.77373 | 0.139796 | -1.35495 | 0.253558 | -2.90328 | 0.006838 |
| DDR2 Y670 | Q16832 | -1.24747 | 0.32215 | -1.21633 | 0.233585 | 1.636214 | 0.085949 | -1.39372 | 0.187038 |
| desmin S11 | P17661 | 1.02094 | 0.480864 | -1.07844 | 0.364181 | 1.073637 | 0.406766 | 1.785982 | 0.077521 |
| desmin T443 | P17661 | -1.47498 | 0.176451 | 1.11903 | 0.38534 | 1.502865 | 0.014761 | 1.158797 | 0.391976 |
| DNAJA1(HDJ2) Y7 | P31689 | -2.36761 | 0.024263 | -1.11178 | 0.137031 | 1.198238 | 0.284069 | 3.459893 | 0.006605 |
| DNCLI1 S197 | Q90828 | -1.17731 | 0.369245 | -1.7947 | 0.064735 | -1.31561 | 0.10717 | -2.97587 | 0.072539 |
| eEF2K S398 | O00418 | 1.965896 | 0.016979 | -1.5738 | 0.10374 | -1.34919 | 0.163185 | -1.96837 | 0.064412 |
| eEF2K S78 | O00418 | 1.034718 | 0.455255 | -1.52822 | 0.099691 | 1.200018 | 0.228668 | 1.858877 | 0.137427 |
| EFNB2 Y304 | P52799 | -1.33596 | 0.074776 | 1.671579 | 0.034765 | 1.904048 | 0.003383 | 1.298424 | 0.224015 |
| ENO1 Y189 | P06733 | -2.41963 | 0.007215 | 1.220142 | 0.179174 | 1.718456 | 0.028327 | 1.766138 | 0.046922 |
| ENO1 Y44 | P06733 | 1.366377 | 0.150038 | -1.23071 | 0.250048 | 1.475618 | 0.077734 | 2.324866 | 0.035187 |
| ENO3 Y124 | P13929 | 2.504269 | 0.00274 | -1.17505 | 0.333995 | -1.38264 | 0.235907 | 2.639674 | 0.012059 |
| EPB41 Y662 | P11171 | -1.80165 | 0.145099 | -1.41727 | 0.205235 | -1.23062 | 0.235309 | 2.138804 | 0.074274 |
| EphA5 Y632 | P54756 | 1.253864 | 0.348477 | -1.55055 | 0.024917 | 1.144883 | 0.377468 | 1.870298 | 0.085631 |
| EphB1 Y594 | P54762 | -1.98861 | 0.056695 | -1.04607 | 0.399435 | -1.82579 | 0.117798 | 1.081092 | 0.39835 |
| EphB1 Y778 | P54762 | 3.149689 | 0.049484 | 1.193947 | 0.283351 | 1.225565 | 0.265277 | -1.22753 | 0.174715 |
| EphB2 Y586 | P29323 | -1.51508 | 0.184498 | 1.030154 | 0.456435 | 1.664684 | 0.012273 | -1.14599 | 0.406321 |
| EphB2 Y605 | P29323 | -2.17526 | 0.000981 | -1.31087 | 0.281772 | 1.142149 | 0.324983 | -1.13515 | 0.294955 |
| EphB3 F618 | P54753 | -1.29349 | 0.113348 | 1.109249 | 0.401294 | -1.01251 | 0.480542 | 1.614301 | 0.168394 |
| EphB4 Y585 | P54760 | -1.07994 | 0.401166 | -1.66045 | 0.212615 | -1.34334 | 0.134076 | 1.502574 | 0.218015 |
| ephexin-1 Y117 | Q8N5V2 | -1.94763 | 0.015502 | 1.268148 | 0.199665 | 1.867822 | 0.094786 | -1.17353 | 0.258775 |
| ephexin-1 Y17 | Q8N5V2 | -1.68617 | 0.012553 | 1.089904 | 0.345807 | 1.471799 | 0.061736 | 1.181481 | 0.332669 |
| ERK3 S189 | Q16659 | -3.41547 | 0.008737 | 1.12911 | 0.26943 | 1.277593 | 0.270405 | 1.62194 | 0.115865 |
| ERK3 T698 | Q16659 | 1.254444 | 0.305257 | -1.56906 | 0.192414 | 1.541592 | 0.106002 | 1.10881 | 0.368421 |
| Ets T38 | P14921 | -1.36695 | 0.176359 | -1.32358 | 0.271619 | -1.20512 | 0.298687 | -1.30898 | 0.306527 |
| FAK Y397 | Q05397 | 1.048076 | 0.393393 | -1.1604 | 0.267959 | 2.06839 | 0.01877 | 1.369557 | 0.275357 |
| FAK Y576 | Q05397 | -1.47754 | 0.090668 | -1.27034 | 0.239112 | 1.20563 | 0.269233 | -1.44843 | 0.087026 |
| FBN1 I2893 | P35555 | -1.19849 | 0.33791 | -2.04013 | 0.009419 | -1.46135 | 0.133389 | 1.550229 | 0.113851 |
| FGF20 T184 | Q9NP95 | -1.37751 | 0.206797 | 2.064123 | 0.019217 | 2.102446 | 0.005645 | 1.086796 | 0.410384 |
| FGFR1OP2 Y48 | Q9NVK5 | 1.793223 | 0.014091 | -1.97472 | 0.021258 | 1.259574 | 0.190247 | -1.18905 | 0.371487 |
| G6PI S184 | P06744 | -1.58308 | 0.056563 | -1.25111 | 0.254456 | -1.16088 | 0.34022 | 1.09167 | 0.372092 |
| G6PI T109 | P06744 | 2.035708 | 0.087755 | -2.13415 | 0.017366 | 1.370467 | 0.243327 | 1.581658 | 0.140233 |
| GADPH Y318 | P04406 | -2.66029 | 0.024542 | -1.09147 | 0.315073 | -1.43776 | 0.161246 | -1.54607 | 0.198898 |
| GADPH Y40 | P04406 | -1.48835 | 0.061735 | 1.031033 | 0.454301 | 1.069371 | 0.435316 | -1.40773 | 0.054597 |
| GDE F29 | P35573 | -1.15862 | 0.287187 | 1.039359 | 0.47266 | -1.21685 | 0.295203 | -1.40666 | 0.0821 |
| GDE Y480 | P35573 | -1.17921 | 0.306368 | -1.17688 | 0.214711 | -1.58578 | 0.04073 | -1.15441 | 0.329025 |
| GDE Y505 | P35573 | -1.28923 | 0.287841 | -1.02985 | 0.452542 | 1.652469 | 0.058015 | -1.19873 | 0.315222 |
| GDE Y585 | P35573 | -1.24103 | 0.278259 | -1.02661 | 0.471824 | -1.11986 | 0.372215 | 1.098751 | 0.331622 |
| GDI1 Y93 | P31150 | -1.98819 | 0.032747 | 1.674913 | 0.096863 | 1.460867 | 0.07223 | 2.090823 | 0.091436 |
| GIT2 Y392 | Q14161 | -1.13036 | 0.322623 | 1.505354 | 0.112734 | -1.03842 | 0.4396 | -2.78577 | 0.00258 |
| Glycogenin Y314 | P46976 | 1.123209 | 0.397285 | 1.317276 | 0.166905 | 1.913764 | 0.112972 | 1.244308 | 0.22301 |
| GPM6A S267 | P51674 | 1.610261 | 0.223602 | -1.12626 | 0.375603 | -1.19787 | 0.268184 | -1.56376 | 0.058198 |
| Grb2 Y209 | P62993 | 1.313021 | 0.210656 | -1.64807 | 0.079109 | -1.28035 | 0.203479 | 1.131343 | 0.373686 |
| GRK5 S484 | P34947 | -1.55259 | 0.095138 | 1.244753 | 0.263024 | 1.130334 | 0.346676 | 2.434582 | 0.063117 |
| GRP78 T227 | P11021 | -2.25781 | 0.007402 | 1.094908 | 0.336803 | -1.02573 | 0.434884 | 1.401705 | 0.205216 |
| GRP94 S346 | P14625 | -1.11161 | 0.368376 | 1.176285 | 0.202869 | -1.05663 | 0.432103 | 1.639469 | 0.193275 |
| GSK3A Y216 | P49840 | -1.61269 | 0.046803 | 1.217133 | 0.211953 | -1.08001 | 0.41537 | 1.11657 | 0.33057 |
| GSK3B S9 | P49841 | -1.11365 | 0.38983 | -1.82734 | 0.010612 | 1.008934 | 0.487385 | 1.846545 | 0.068158 |
| GTPBP4 Y38 | Q9BZE4 | -1.41603 | 0.010645 | 1.593255 | 0.163338 | 1.692949 | 0.121531 | 1.129179 | 0.366788 |
| H1R T474 | P35367 | 1.07951 | 0.376353 | -1.21398 | 0.239591 | 1.006683 | 0.492181 | 1.303646 | 0.264544 |
| HBS1 Y437 | Q9Y450 | -1.88166 | 0.042815 | 1.298227 | 0.076063 | 1.115411 | 0.410718 | 2.782892 | 0.036967 |
| HK2 Y301 | P52789 | 1.804411 | 0.008809 | 1.287923 | 0.214455 | 1.332963 | 0.183313 | 2.149935 | 0.047853 |
| HSC70 T265 | P11142 | 3.033953 | 0.007341 | 1.070159 | 0.399396 | -1.45915 | 0.194816 | 1.227247 | 0.333717 |
| HSC70 Y41 | P11142 | -1.56739 | 0.105907 | 1.300119 | 0.171916 | -1.00685 | 0.485176 | 1.346668 | 0.213198 |
| HSP60 Y227 | P10809 | 1.553203 | 0.124976 | 1.070607 | 0.411388 | -1.33134 | 0.165333 | 1.390067 | 0.115975 |
| HSP70 Y525 | NP 005336 | 1.305336 | 0.235717 | -1.545 | 0.023891 | -1.19769 | 0.306617 | -1.14221 | 0.344572 |
| HSP70RY S76 | P34932 | 1.163268 | 0.299942 | 1.355899 | 0.318851 | 1.540256 | 0.109139 | -2.8708 | 0.027428 |
| HSP70RY Y336 | P34932 | -1.68233 | 0.114227 | 1.454743 | 0.095645 | -1.29686 | 0.21026 | -1.22873 | 0.240819 |
| HSP90AB2P Y485 | Q58FF8 | 1.09212 | 0.423496 | 1.056218 | 0.433527 | -1.13032 | 0.356117 | 1.533546 | 0.114257 |
| HSP90B S226 | P08238 | 1.315703 | 0.329058 | -1.22612 | 0.293406 | -1.6727 | 0.08002 | 1.370311 | 0.237444 |
| HSP90B Y485 | P08238 | -1.40616 | 0.175965 | 1.334416 | 0.132937 | 1.106907 | 0.295368 | -1.09308 | 0.332323 |
| HSPA1L T505 | P34931 | 1.297726 | 0.280847 | -2.16201 | 0.036398 | -1.13218 | 0.386879 | -1.40075 | 0.238412 |
| HSPA4L T764 | O95757 | -1.08584 | 0.361066 | -1.35948 | 0.162852 | 1.118002 | 0.344409 | 1.298918 | 0.103012 |
| HSPA4L Y630 | O95757 | -1.44226 | 0.125091 | -1.0592 | 0.361137 | 1.282263 | 0.245344 | -1.2652 | 0.218543 |
| HSPA9B T64 | P38646 | -1.1491 | 0.303485 | 1.329819 | 0.093781 | 1.031319 | 0.457209 | -1.40852 | 0.240799 |
| HuR S158 | Q15717 | -1.38729 | 0.132277 | -1.15574 | 0.248548 | 1.545398 | 0.111513 | 1.112363 | 0.362486 |
| HuR S221 | Q15717 | -1.39227 | 0.093098 | 2.410536 | 0.006038 | 2.119288 | 0.02302 | 2.278308 | 0.054554 |
| IKK-epsilon S172 | Q14164 | -1.29633 | 0.120681 | -1.13111 | 0.290725 | 1.683053 | 0.094776 | 1.244307 | 0.264861 |
| IL1RAPL2 S332 | Q9NP60 | 1.198421 | 0.249167 | -1.83606 | 0.050837 | 1.032042 | 0.453507 | 2.007309 | 0.070985 |
| IMP-1 S12 | Q9NZI8 | 1.069291 | 0.398929 | -1.30339 | 0.139459 | 1.056408 | 0.391557 | -1.01271 | 0.48511 |
| IQGAP1 Y536 | P46940 | -1.03899 | 0.456661 | -1.05941 | 0.364425 | -1.09917 | 0.340464 | -1.21283 | 0.292461 |
| IRS-1 Y895 | P35568 | -1.4444 | 0.074018 | -1.01753 | 0.46856 | -1.47193 | 0.252207 | 1.381732 | 0.135467 |
| IRSp53 T363 | Q9UQB8 | 2.445158 | 0.030063 | 1.667409 | 0.020112 | -1.05096 | 0.467501 | 1.043035 | 0.425208 |
| LDH-A Y239 | P00338 | -1.26438 | 0.260848 | -1.36522 | 0.244142 | 1.018438 | 0.479028 | 1.505046 | 0.1925 |
| LDH-B T248 | P07195 | -3.00073 | 0.033449 | 1.117071 | 0.372704 | 1.076635 | 0.417304 | 1.378166 | 0.0981 |
| LIPI Y395 | Q6XZB0 | -1.95393 | 0.004394 | 1.365045 | 0.171475 | 1.07051 | 0.38132 | 1.187531 | 0.342798 |
| LKB1 S428 | Q15831 | -1.71154 | 0.067766 | -1.20814 | 0.272794 | -1.46407 | 0.154752 | -1.09273 | 0.401828 |
| LKB1 T185 | Q15831 | -1.1951 | 0.364101 | -1.30696 | 0.203399 | -1.0927 | 0.316406 | 1.065724 | 0.45345 |
| LKB1 T365 | Q15831 | -1.92679 | 0.036753 | -1.07149 | 0.402389 | -1.34354 | 0.077454 | -1.24476 | 0.24435 |
| MAP1B S1410 | P46821 | -2.13832 | 0.031219 | -1.76229 | 0.143168 | 1.056391 | 0.440877 | 3.596574 | 0.012169 |
| MAPKAPK3 T204 | Q16644 | 1.016982 | 0.476193 | -1.06894 | 0.426508 | 1.156112 | 0.319232 | -1.5543 | 0.111334 |
| MARCKS S128 | P29966 | -1.25993 | 0.193931 | 1.26811 | 0.208691 | 1.031766 | 0.449324 | 1.213785 | 0.295026 |
| MARK1 T215 | Q9P0L2 | 1.022803 | 0.472615 | -1.70799 | 0.093883 | 1.169626 | 0.268713 | 1.520675 | 0.171673 |
| MARK3 T211 | P27448 | -1.62511 | 0.148285 | -1.11756 | 0.339907 | 2.82946 | 0.001479 | 1.275204 | 0.267791 |
| MAT2A Y102 | P31153 | -1.59787 | 0.059878 | 1.357923 | 0.179288 | 1.775086 | 0.045359 | 1.510857 | 0.079177 |
| MC4R T311 | P32245 | 2.97386 | 0.010874 | -1.64271 | 0.036135 | 1.057314 | 0.42793 | 1.842594 | 0.090855 |
| MDM2 S241 | Q00987 | -1.4405 | 0.171031 | -1.01658 | 0.47984 | 1.584 | 0.048603 | 1.776444 | 0.036982 |
| MEK1 S211 | Q02750 | -1.31779 | 0.283741 | -2.39162 | 0.029141 | -1.16154 | 0.355862 | 1.306102 | 0.285684 |
| MEK2 S304 | P36507 | 1.659191 | 0.09231 | -1.13978 | 0.326665 | -1.98569 | 0.069313 | 1.434755 | 0.148463 |
| MEK5 S234 | Q13163 | -1.36686 | 0.208315 | -1.04011 | 0.446096 | 1.352102 | 0.121683 | -1.0354 | 0.467414 |
| MEKK1 S131 | Q13233 | 1.271505 | 0.254685 | 1.602415 | 0.039603 | -1.36217 | 0.196449 | -1.18588 | 0.313002 |
| MEKK3 S197 | Q99759 | 2.588657 | 0.029773 | 1.222396 | 0.30691 | -1.20372 | 0.405302 | -1.61281 | 0.047241 |
| MEKK3 S551 | Q99759 | -1.14715 | 0.302459 | 1.048644 | 0.407319 | 1.209398 | 0.343806 | 1.429902 | 0.050188 |
| MEKK3 T321 | Q99759 | 1.679968 | 0.130361 | -1.15815 | 0.317612 | -1.0735 | 0.395565 | 1.024553 | 0.466235 |
| Met Y1235 | P08581 | 1.410336 | 0.211391 | 1.279842 | 0.233085 | -1.58638 | 0.059942 | -1.21118 | 0.280164 |
| Met Y1357 | P08581 | -1.26743 | 0.184289 | -1.02432 | 0.460988 | -1.64683 | 0.052317 | 1.08655 | 0.370658 |
| mGluR1 Y664 | Q13255 | 1.281631 | 0.294076 | -1.41076 | 0.189477 | 1.07518 | 0.38776 | 1.763195 | 0.174048 |
| MKK3 S129 | P46734 | -1.9151 | 0.11937 | -1.1518 | 0.356653 | -1.64065 | 0.158594 | -1.08358 | 0.381794 |
| MKK4 S44 | P45985 | 2.037429 | 0.029664 | -2.89182 | 0.005979 | -1.45727 | 0.144927 | -1.28622 | 0.209935 |
| MKK4 T225 | P45985 | 2.883825 | 0.039509 | -1.15954 | 0.350751 | -1.6466 | 0.10014 | -6.15776 | 0.006998 |
| MKK6 S129 | P52564 | 2.083349 | 0.007175 | -2.41946 | 0.006051 | -1.50263 | 0.193474 | -2.50291 | 9.48E-06 |
| MKP-1 S298 | P28562 | 1.402402 | 0.116571 | -1.55115 | 0.186208 | -1.45012 | 0.196525 | -1.58784 | 0.074869 |
| MKP-3 S178 | Q16828 | -1.10656 | 0.33829 | -1.45211 | 0.121947 | -1.20079 | 0.240203 | -1.34374 | 0.161294 |
| MKP-5 S4 | Q9Y6W6 | 1.957332 | 0.028827 | 1.15057 | 0.300437 | -1.05551 | 0.419594 | -2.46421 | 0.028402 |
| MLK3 Y269 | Q16584 | 2.286065 | 0.035027 | -1.94505 | 0.007263 | 1.071981 | 0.429335 | 1.145287 | 0.27677 |
| MOR1 S312 | P35372 | 1.559486 | 0.071925 | -2.59403 | 0.003572 | 1.159361 | 0.346248 | -1.23538 | 0.250409 |
| MOR1 Y146 | P35372 | 1.600383 | 0.037372 | -1.07793 | 0.404721 | 1.028774 | 0.467566 | -1.26159 | 0.197638 |
| MTOR S2366 | P42345 | 1.100326 | 0.329477 | -1.34321 | 0.185727 | -1.57738 | 0.042845 | -1.45049 | 0.200292 |
| MTOR S2399 | P42345 | -1.47444 | 0.045853 | 1.027388 | 0.464461 | 1.088115 | 0.421932 | 1.303335 | 0.216247 |
| MYPT1 T695 | O14974 | 1.251339 | 0.17918 | -1.00565 | 0.490124 | -1.27122 | 0.320948 | -1.58566 | 0.01093 |
| MYPT1 T850 | O14974 | -1.01629 | 0.47239 | 1.248301 | 0.254302 | 1.124379 | 0.205127 | -1.01619 | 0.472877 |
| nAChRA7 Y442 | P36544 | -1.77988 | 0.056823 | 1.108934 | 0.362147 | 1.137444 | 0.383199 | 1.02028 | 0.432633 |
| NCAM Y1258 | P32004 | 2.260968 | 0.062841 | -1.49855 | 0.145573 | -1.3463 | 0.235667 | -1.66552 | 0.068788 |
| NCAPH S375 | Q15003 | 1.183226 | 0.223005 | 1.006231 | 0.492104 | -1.29648 | 0.138085 | 1.103134 | 0.351964 |
| NCX1 Y258 | P32418 | -1.17659 | 0.249767 | -1.32709 | 0.231239 | -1.91065 | 0.027646 | -1.75815 | 0.013085 |
| NFAT1 S761 | Q13469 | 1.466248 | 0.046987 | -1.06473 | 0.432726 | -1.06906 | 0.440159 | -1.27032 | 0.29011 |
| NFIL3 S283 | Q16649 | 1.207736 | 0.172553 | 1.040035 | 0.444608 | -1.10888 | 0.401454 | 1.337587 | 0.221855 |
| NGFR S292 | P08138 | 1.33961 | 0.221615 | -1.19232 | 0.294177 | -2.09868 | 0.047318 | -1.06335 | 0.430344 |
| NKEFB T96 | P32119 | 1.313082 | 0.294411 | 1.330761 | 0.20756 | 1.030918 | 0.480409 | 1.147182 | 0.311707 |
| Nuak1 S614 | O60285 | 1.09885 | 0.363144 | -2.06488 | 0.049106 | -1.0477 | 0.443844 | -2.18441 | 0.023129 |
| Nuak1 T225 | O60285 | -1.61447 | 0.134357 | 1.335094 | 0.15407 | 1.084063 | 0.382773 | 1.190573 | 0.220175 |
| p38-alpha Y182 | Q16539 | -1.44576 | 0.043782 | -1.23763 | 0.164228 | 1.292809 | 0.183164 | 1.92524 | 0.060724 |
| p70S6K T389 | P23443 | 1.052497 | 0.432764 | 1.103977 | 0.195661 | 1.194127 | 0.338411 | 1.481779 | 0.056326 |
| P90RSK S398 | Q15418 | 1.292531 | 0.242594 | 1.080455 | 0.389792 | 1.36642 | 0.117586 | -1.54465 | 0.046441 |
| PAK1 S198 | Q13153 | 1.306836 | 0.175658 | -1.29679 | 0.169217 | -1.37217 | 0.238338 | -1.30099 | 0.203535 |
| PAK1 T422 | Q13153 | -1.03727 | 0.455453 | -1.26297 | 0.129897 | 2.186784 | 0.113215 | -1.22102 | 0.11426 |
| PAK2 S195 | Q13177 | 2.578375 | 0.017935 | -2.45995 | 0.011953 | 1.242873 | 0.261929 | -1.62295 | 0.144576 |
| PDK1 S244 | O15530 | -1.149 | 0.333962 | -1.12217 | 0.333129 | -1.1354 | 0.379381 | -1.15576 | 0.391982 |
| PFKFB1 S31 | P16118 | -1.18781 | 0.317767 | 1.111092 | 0.36801 | 1.322365 | 0.121216 | 1.334046 | 0.110652 |
| PFKFB2 S467 | O60825 | 1.39155 | 0.058078 | 1.403309 | 0.157544 | 1.002601 | 0.494933 | -1.03474 | 0.459891 |
| PFKFB2 Y364 | O60825 | 1.005223 | 0.494653 | 1.31376 | 0.204515 | 1.236776 | 0.270616 | -1.18593 | 0.278602 |
| PFKFB3 S462 | Q16875 | 1.247992 | 0.209265 | 1.671555 | 0.169919 | -1.05591 | 0.417244 | -1.94884 | 0.080874 |
| PFKFB3 S462 | Q16875 | 1.352365 | 0.205255 | 2.08907 | 0.051775 | -1.07492 | 0.442628 | -1.04309 | 0.460437 |
| PFKM S365 | P08237 | -1.47065 | 0.092543 | 1.976667 | 0.042815 | 1.920189 | 0.002705 | 1.461 | 0.205471 |
| PGAM1 C23 | P18669 | 1.259342 | 0.211662 | 1.09514 | 0.364526 | -1.008 | 0.48824 | 1.067728 | 0.421091 |
| PGAM1 S14 | P18669 | 1.588984 | 0.096111 | 1.231322 | 0.262842 | 1.229344 | 0.286505 | -1.44826 | 0.061415 |
| PGAM2 Y92 | P15259 | -1.47873 | 0.067703 | 1.965807 | 0.033116 | -1.73112 | 0.063567 | 2.321981 | 0.03378 |
| PGAM4 S118 | Q8N0Y7 | -1.1691 | 0.293245 | -1.05777 | 0.42662 | 1.058374 | 0.403443 | 1.118529 | 0.439504 |
| PGC-1 S264 | Q9UBK2 | 1.520871 | 0.084955 | -1.62028 | 0.1106 | 1.021244 | 0.480846 | -2.46799 | 0.03419 |
| PGK1 Y196 | P00558 | 1.53196 | 0.165804 | -1.10257 | 0.360337 | 1.164468 | 0.239014 | -1.2498 | 0.259507 |
| PGM1 T467 | P36871 | 1.020928 | 0.475625 | -2.06194 | 0.026321 | -1.23292 | 0.132899 | -1.08674 | 0.405746 |
| PGM1 Y354 | P36871 | 1.268367 | 0.257532 | -2.24149 | 0.094973 | -1.06165 | 0.422699 | -1.84618 | 0.054377 |
| PGM2 Y565 | Q96G03 | 1.194257 | 0.254718 | -1.18964 | 0.265514 | -1.36286 | 0.214943 | -1.05591 | 0.416308 |
| PGM2L1 S162 | Q6PCE3 | 2.196592 | 0.005568 | -1.16659 | 0.358938 | -1.55409 | 0.164053 | -1.76466 | 0.0447 |
| PGM3 S64 | O95394 | 1.155827 | 0.210968 | -1.42903 | 0.131771 | -1.46649 | 0.212792 | 1.108063 | 0.381605 |
| PHB Y114 | P35232 | 1.052941 | 0.424997 | -2.0854 | 0.046117 | 1.068306 | 0.432635 | -1.53763 | 0.197414 |
| PHKA1 S967 | P46020 | -1.03048 | 0.453049 | 1.089841 | 0.350424 | -1.32334 | 0.256343 | -1.52405 | 0.061758 |
| PHKA1 Y549 | P46020 | 1.053704 | 0.459554 | 1.655936 | 0.083666 | -1.10979 | 0.432384 | -1.77206 | 0.00258 |
| PHKA2 S727 | P46019 | 1.495387 | 0.131358 | 1.66377 | 0.041197 | 1.162687 | 0.294249 | -1.47562 | 0.165266 |
| PHKB S694 | Q93100 | 1.836588 | 0.067598 | 1.175766 | 0.2438 | -1.285 | 0.282023 | -2.7698 | 0.009077 |
| PHKG1 S31 | Q16816 | -1.29326 | 0.210761 | -1.7052 | 0.141988 | -2.85193 | 0.014128 | -1.1038 | 0.35402 |
| PHKG1 Y337 | Q16816 | 1.970913 | 0.029063 | -1.1279 | 0.402942 | -1.56767 | 0.071504 | -1.69592 | 0.079603 |
| PIK3R1 Y467 | P27986 | 1.473077 | 0.084185 | -1.13501 | 0.343649 | -1.08355 | 0.413465 | 1.132232 | 0.369688 |
| PIK3R1 Y528 | P27986 | -2.05735 | 0.007667 | -1.27401 | 0.258932 | 1.075852 | 0.430279 | 2.601102 | 0.014972 |
| PIK3R1 Y556 | P27986 | 1.250874 | 0.292715 | 1.040654 | 0.461489 | -1.53463 | 0.084843 | -1.71357 | 0.07472 |
| PKACa S187 | P17612 | -1.32207 | 0.156884 | 1.227589 | 0.320239 | -1.4454 | 0.246587 | 1.202457 | 0.410775 |
| PKACa T245 | P17612 | 1.09921 | 0.352511 | -1.3118 | 0.204683 | -1.88213 | 0.012061 | 1.547568 | 0.252592 |
| PKAR1A S83 | P10644 | -1.09671 | 0.396852 | -2.52842 | 0.008122 | 1.073057 | 0.455113 | -1.89868 | 0.009178 |
| PKAR1B Y312 | P31321 | 1.176103 | 0.296551 | -1.37323 | 0.073769 | 1.142658 | 0.328598 | -1.47745 | 0.026223 |
| PKAR2B S115 | P31323 | 2.127404 | 0.020693 | -4.08203 | 0.003475 | -1.32872 | 0.270131 | 1.004838 | 0.492923 |
| PKCA S659 | P17252 | 1.050355 | 0.458868 | 1.199698 | 0.207571 | 1.197675 | 0.307494 | 1.373 | 0.090629 |
| PKCA T499 | P17252 | 1.051378 | 0.408449 | 1.296439 | 0.2182 | -1.07917 | 0.427552 | 1.429277 | 0.130893 |
| PKCA T640 | P17252 | 1.740047 | 0.00683 | -1.56748 | 0.127845 | 1.646047 | 0.074871 | -1.33271 | 0.114746 |
| PKCD Y185 | Q05655 | 1.140217 | 0.327447 | -1.2078 | 0.277697 | 1.920961 | 0.101301 | -1.92485 | 0.069356 |
| PKCD Y64 | Q05655 | 1.223524 | 0.287102 | 1.203039 | 0.250371 | -1.7666 | 0.061545 | 1.342746 | 0.114775 |
| PKCT T216 | Q04759 | -1.14587 | 0.379238 | -1.1142 | 0.353119 | 1.156324 | 0.343199 | -1.29037 | 0.247586 |
| PKD1 Y442 | Q15139 | 1.222354 | 0.342246 | 1.030109 | 0.450908 | -1.42856 | 0.153095 | 1.204683 | 0.235019 |
| PKLR T556 | P30613 | -1.0955 | 0.399623 | 1.171099 | 0.368681 | -1.10344 | 0.36716 | -1.60058 | 0.081072 |
| PKM2 Y280 | P14618 | 1.210949 | 0.309484 | 1.053748 | 0.447759 | -1.19276 | 0.336843 | -1.83656 | 0.006349 |
| PLCB1 Y1310 | Q9NQ66 | -1.24608 | 0.256835 | -1.20403 | 0.338935 | -1.50346 | 0.233713 | -1.27007 | 0.202355 |
| PMCA1 S1153 | P20020 | -2.18035 | 0.006014 | 1.06706 | 0.437369 | 1.203746 | 0.261457 | 1.645719 | 0.109017 |
| profilin2 Y99 | P35080 | 1.069928 | 0.336314 | -1.11502 | 0.395731 | -1.59306 | 0.075441 | 1.132517 | 0.328488 |
| PTEN S346 | P60484 | -1.63578 | 0.088966 | 1.646122 | 0.125722 | -1.48288 | 0.039715 | -1.06055 | 0.448503 |
| PTEN Y206 | P60484 | -1.79461 | 0.080767 | 1.159283 | 0.283922 | 1.400462 | 0.181169 | 1.112519 | 0.40096 |
| PTP1B Y152 | P18031 | -1.74638 | 0.01483 | -1.4235 | 0.22088 | -1.7558 | 0.004198 | -3.08348 | 0.011613 |
| PXN S273 | P49023-2 | -1.18877 | 0.287751 | -1.27124 | 0.238616 | 1.07168 | 0.406866 | -1.49502 | 0.128653 |
| PYGL Y186 | P06737 | -1.89926 | 0.022984 | -1.36063 | 0.103799 | 1.217381 | 0.355464 | 1.6786 | 0.031686 |
| PYGM Y733 | P11217 | -1.16576 | 0.315439 | 1.200755 | 0.181485 | -1.15873 | 0.309658 | -1.03188 | 0.45496 |
| PYK2 Y577 | Q14289 | 1.024311 | 0.467281 | -1.88128 | 0.111255 | 1.648467 | 0.034508 | 1.011153 | 0.490644 |
| Raf1 S259 | P04049 | 1.628573 | 0.130229 | -1.57643 | 0.103786 | -1.21859 | 0.200774 | -1.9755 | 0.027062 |
| Raf1 S338 | P04049 | 1.036521 | 0.458273 | 1.257041 | 0.149834 | -1.21838 | 0.301474 | 1.152796 | 0.299722 |
| RASGEF1B Y86 | Q96MY8 | -1.00807 | 0.491974 | 1.482639 | 0.172036 | 1.200986 | 0.325209 | 1.527473 | 0.175357 |
| RasGRP3 T133 | Q8IV61 | 2.148818 | 0.004694 | 1.078594 | 0.421716 | -1.89341 | 0.06915 | 1.287066 | 0.142868 |
| RASSF5 T187 | Q8WWW0 | -1.06967 | 0.419535 | -1.32376 | 0.190417 | -1.32825 | 0.240005 | 1.179432 | 0.348263 |
| RASSF8 S105 | Q8NHQ8 | 1.054756 | 0.443399 | 1.082761 | 0.39783 | -1.03484 | 0.457933 | 1.181615 | 0.242628 |
| RSK2 S396 | P51812 | 1.496274 | 0.142933 | 1.101655 | 0.325523 | -1.5761 | 0.217722 | 1.529861 | 0.100927 |
| SCAMP4 Y205 | Q969E2 | 1.280814 | 0.20507 | 1.779512 | 0.044609 | -1.44698 | 0.085768 | -1.17498 | 0.236044 |
| SDHA Y366 | P31040 | 1.288708 | 0.222881 | -1.09706 | 0.384206 | -1.87097 | 0.083231 | 1.277353 | 0.157135 |
| SERT T316 | P31645 | -1.4979 | 0.107137 | 1.311936 | 0.256425 | 1.451006 | 0.054746 | 1.631531 | 0.055721 |
| SGEF Y279 | Q96DR7 | -1.87363 | 0.020351 | -1.27086 | 0.23687 | -1.4992 | 0.054424 | 1.042768 | 0.458699 |
| SirT1 S184 | Q96EB6 | 1.42889 | 0.251344 | -1.51504 | 0.182527 | -1.07163 | 0.408818 | -1.53162 | 0.057426 |
| smMLCK S1749 | Q15746 | 2.52203 | 0.047142 | -1.62869 | 0.176597 | -1.18367 | 0.373692 | -2.20173 | 0.013647 |
| SOLH T13 | O75808 | -1.09515 | 0.422217 | -2.00489 | 0.039779 | -1.1257 | 0.351475 | 2.422585 | 0.063746 |
| SRC-1 S101 | Q15788 | 1.101258 | 0.375794 | -1.88249 | 0.064988 | -1.95216 | 0.051993 | -3.14625 | 0.000434 |
| SREBP-1 S314 | P36956 | 1.739583 | 0.018377 | -4.29527 | 0.003233 | -1.21588 | 0.257559 | -1.2402 | 0.262534 |
| ST13 S77 | P50502 | 1.13465 | 0.354943 | -1.76469 | 0.044094 | -1.64936 | 0.135632 | -3.4358 | 0.000471 |
| TAB1 S423 | Q15750 | 1.130912 | 0.367873 | -2.77324 | 0.034817 | -1.19247 | 0.315136 | -1.98842 | 0.0347 |
| TGFBR1 T200 | P36897 | -1.20801 | 0.223393 | -1.25081 | 0.280071 | -1.35961 | 0.16962 | -1.11893 | 0.351019 |
| TMEFF2 Y166 | Q9UIK5 | 1.149574 | 0.324579 | -1.62709 | 0.082172 | 1.426936 | 0.13174 | -1.24274 | 0.088207 |
| TNIK S678 | Q9UKE5 | 1.810193 | 0.010613 | 1.193432 | 0.326152 | -1.66325 | 0.122802 | -2.74555 | 0.000115 |
| TPI1 Y164 | P60174 | 1.025194 | 0.448014 | 1.405705 | 0.210454 | -1.46756 | 0.19527 | 1.582419 | 0.045646 |
| TRAF6 Y355 | Q9Y4K3 | -1.52636 | 0.120033 | -1.43453 | 0.137648 | 1.075768 | 0.403818 | 2.4535 | 0.008398 |
| TRAF7 S88 | Q6Q0C0 | 1.389868 | 0.067037 | 1.334602 | 0.206421 | -1.02804 | 0.475268 | 2.117471 | 0.013676 |
| TSC1 T393 | Q92574 | -1.03031 | 0.446873 | -1.5705 | 0.108639 | -1.577 | 0.08588 | -1.26254 | 0.134674 |
| UQCRC1 Y434 | P31930 | -1.5671 | 0.143759 | 1.164024 | 0.304371 | -1.37755 | 0.217793 | 1.102526 | 0.403287 |
| utrophin Y2573 | P46939 | -1.08451 | 0.340786 | 1.008795 | 0.489499 | -1.97713 | 0.046101 | -3.69529 | 2.91E-05 |
| vinculin Y1134 | P18206 | -1.00586 | 0.490949 | 1.391706 | 0.193587 | -1.48249 | 0.211417 | -1.74216 | 0.039595 |
| vinculin Y822 | P18206 | -1.74043 | 0.021983 | -1.66191 | 0.083224 | 1.155512 | 0.317734 | 1.175409 | 0.137565 |
